# Biased competition between Lgr5 intestinal stem cells driven by oncogenic mutation induces clonal expansion

**DOI:** 10.1002/embr.201337799

**Published:** 2013-12-16

**Authors:** Hugo J Snippert, Arnout G Schepers, Johan H van Es, Benjamin D Simons, Hans Clevers

**Affiliations:** 1 Hubrecht Institute, KNAW & University Medical Center UtrechtUtrecht, the Netherlands; 2 Molecular Cancer Research, University Medical Center UtrechtUtrecht, the Netherlands; 3 Harvard-MIT, Division of Health Sciences and Technology, Massachusetts Institute of TechnologyCambridge, MA, USA; 4 Cavendish Laboratory, Department of Physics, University of CambridgeCambridge, UK; 5 The Wellcome Trust/ Cancer Research UK Gurdon Institute, University of CambridgeCambridge, UK; 6 Wellcome Trust-Medical Research Council Stem Cell Institute, University of CambridgeCambridge, UK

**Keywords:** crypt fission, field cancerization, K-ras, Lgr5 intestinal stem cells, neutral competition

## Abstract

The concept of ‘field cancerization’ describes the clonal expansion of genetically altered, but morphologically normal cells that predisposes a tissue to cancer development. Here, we demonstrate that biased stem cell competition in the mouse small intestine can initiate the expansion of such clones. We quantitatively analyze how the activation of oncogenic *K-ras* in individual Lgr5^+^ stem cells accelerates their cell division rate and creates a biased drift towards crypt clonality. *K-ras* mutant crypts then clonally expand within the epithelium through enhanced crypt fission, which distributes the existing Paneth cell niche over the two new crypts. Thus, an unequal competition between wild-type and mutant intestinal stem cells initiates a biased drift that leads to the clonal expansion of crypts carrying oncogenic mutations.

## Introduction

The adenoma-carcinoma sequence of colorectal cancers (CRCs), defined histopathologically, has been postulated to occur as the consequence of an ordered series of mutations in a limited set of cancer genes (e.g. *APC*,*KRAS*,*P53, Smad4*) 1. There is a consensus that *APC* mutations initiate intestinal neoplasias. *KRAS* mutations are believed to play an important role in progression towards adenocarcinomas 1. Yet, a priori, there is no reason to expect that these mutations must be acquired in this order 2. For instance, oncogenic mutations in *K-ras* have been detected in histologically normal epithelium that surrounded resected colorectal cancers of patients 3 4. For a wide variety of epithelial cancers, clinical evidence accumulates that cancer development can start with the clonal expansion of mutant cell clones that, although histologically normal, predispose the tissue for subsequent tumor growth 5.

The small intestinal epithelium of mice provides an attractive model system to study adult stem cell biology and the role of stem cells in cancer development due to its structural organization of proliferating and differentiated cells 6. Approximately 16 proliferative ‘Crypt Base Columnar’ (CBC) cells, representing the Lgr5^+^ stem cells of the intestine, are present at the base of each crypt, optimally distributed between Paneth cells that, together with the surrounding mesenchyme, constitute the stem cell niche 7 8 9. The fate of intestinal stem cells is determined through neutral competition for niche occupancy. Stem cells that become displaced from Paneth cell contact lose stemness and enter the transit amplifying (TA) compartment. As a result, clones within the niche can either expand or contract. Eventually, one clone will outcompete all other stem cell clones, thus rendering the crypt monoclonal 7 10 (supplementary Fig S1).

Using mouse models, deletion of APC, or constitutive activation of oncogenic β-catenin in the Lgr5 stem cell compartment of the small intestine identified them as cells-of-origin of intestinal neoplasia 11 12. Moreover, the Lgr5^+^ cell population within existing intestinal adenomas maintain stem cell activity and fuels the growth of the tumor 13. Although oncogenic mutated *K-ras* that is driven from the endogenous locus induces hyperplasia in a variety of tissues, including the colon, no morphologically detectable abnormalities are observed in the proximal small intestine 14 15 16 17 18 (supplementary information), despite its role in progressing intestinal adenomas towards a more aggressive adenomacarcinoma 16.

The term ‘field cancerization’ was first proposed by Slaughter *et al* in 1953 19. Currently, it is used to describe clonally expanding fields of genetically altered, but histologically normal cells that predispose tissues for cancer development 20. Despite increasing clinical recognition and evidence, underlying processes that initiate expansion of such clones are not well understood 21. Here, upon sporadic activation of oncogenic K-ras, we provide insights into how an unequal competition between intestinal stem cells initiates a biased drift to crypt clonality that is followed by clonal expansion through enhanced crypt fission.

## Results and Discussion

### Clonal expansion of K-ras mutated stem cells

**Figure 1:**
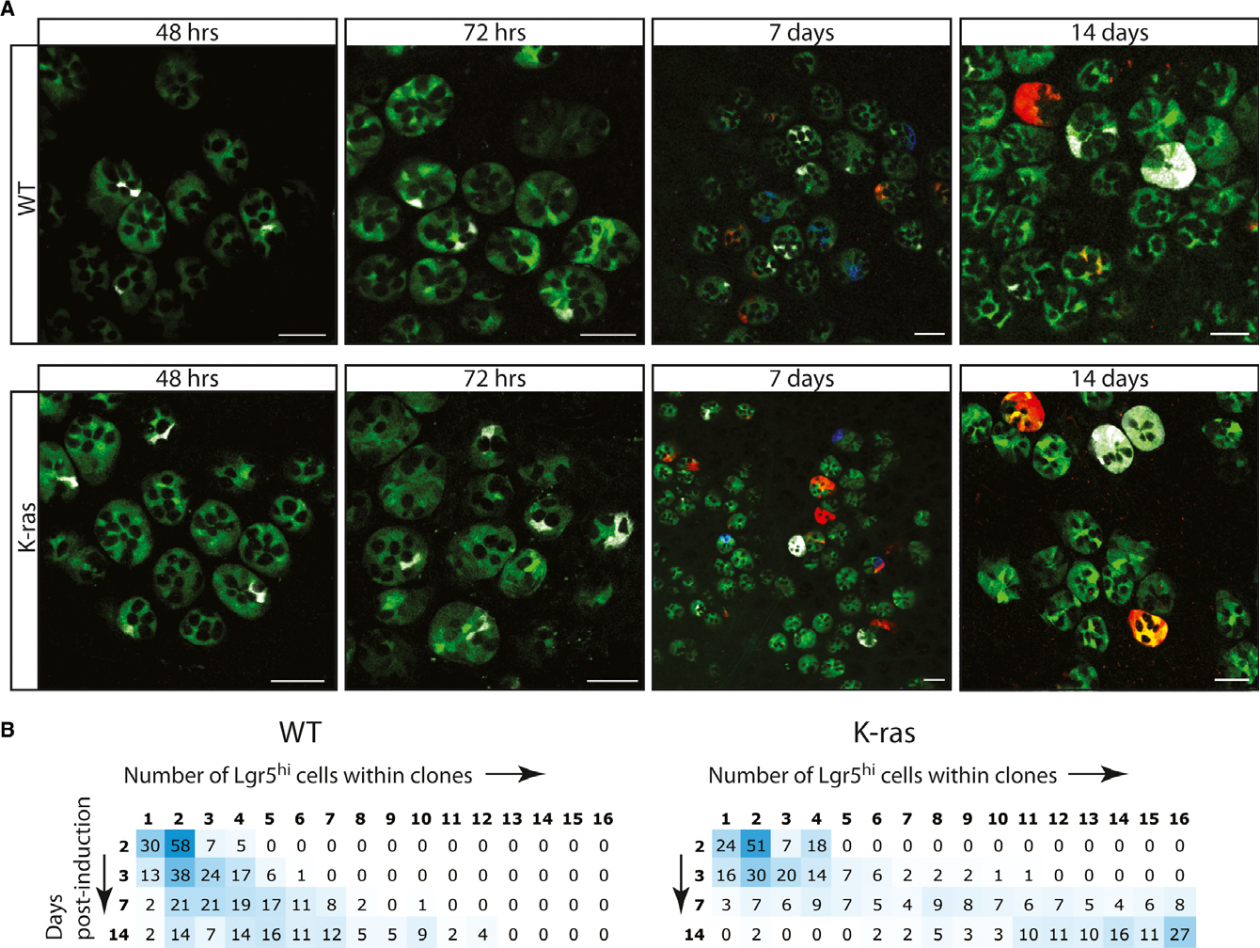
Clonal expansion of sporadically induced K-ras^G12D^ in Lgr5^hi^ cells Confocal scanning of the bottom of small intestinal crypts at indicated time points after sporadic activation of K-ras^G12D^ mutation in intestinal stem cells (bottom panels) or in WT controls (top panels). Lgr5 stem cells are marked with EGFP (green). Clones are randomly marked with YFP (pseudo color white), RFP (red) or membrane tagged CFP (blue), driven from the R26R-Confetti locus. K-ras^G12D^ clones expand faster over time than their WT counterparts, many crypts being fixated within 14 days of tracing. Scale bars; 50 μm.Expansion of Lgr5^hi^ cell numbers over time within clones that contain at least one Lgr5^hi^ cell. The numbers represent the percentage of clones with a certain number of Lgr5^hi^ cells for each time point. As the average size of clones gradually increases in WT, the K-ras^G12D^ activated clones colonize the stem cell compartment much faster. Blue hues represent the relative frequency of Lgr5^hi^ cell numbers per time point, 50% is blue; 0% is white. Left matrix is for WT clones, right matrix is for K-ras^G12D^ clones. Data information: Data from WT clones is reproduced from 7.

To investigate the effect of an oncogenic mutation on intestinal stem cell behavior, we sporadically activated oncogenic K-ras^G12D^ in Lgr5^+^ intestinal stem cells, whose fate could be followed via the simultaneous activation of the multicolor Cre-reporter *R26R-Confetti* (supplementary information). Thereby we created a mosaic situation of WT stem cells with a few marked mutant stem cells. There was no obvious difference in clone density (number of clones per unit area of tissue) between K-ras^G12D^ and WT ‘Confetti’ clones indicating that the induction efficiency was comparable (Fig1A). A subtle difference in clone size appeared after 72 h of tracing. On average, clones in K-ras mice contained more cells than WT (supplementary Fig S2). This effect became more pronounced after 7 and 14 days of tracing. At these time points, a significant frequency of ‘clonal fixations’ (i.e. crypts in which all stem cells belong to the same clone) was observed in K-ras mice, a feature never seen in WT (Fig1B).

Next, we quantified the size and stem cell content of clones. After 48 h of tracing, the average size of K-ras clones was almost identical to WT, although the weight was slightly skewed towards higher stem cell number (supplementary Fig S2A). After 72 h of tracing, K-ras clones were significantly larger than WT, and contained proportionately more Lgr5^hi^ stem cells. Moreover, clonal ‘extinction’ (i.e. complete loss of stem cells within a marked clone) occurred less frequently in K-ras mice (21%) compared to WT (34%), suggesting that K-ras mutant stem cells have a survival advantage over their WT neighbors (supplementary Fig S2).

By 1 week of tracing, many of the clonal progeny had migrated into the villus compartment, outside the microscope detection range, making it impossible to score complete clone sizes. Nevertheless, the expansion of clones within the stem cell population could be traced. To focus on a defined population, we restricted our analysis to ‘surviving’ clones, defined as those that retain at least one Lgr5^hi^ stem cell and noticed that the average stem cell content in surviving K-ras clones increased much faster over time than that of WT (Fig1B).

### Clonal expansion as a result of competitive advantage

Previous studies have shown that the model of neutral drift dynamics can be used to develop quantitative insights into the pattern of clonal evolution in WT tissue. In particular, labeled clones in the stem cell compartment expand or contract in a ‘random walk’-type process at a rate set by the frequency of loss (displacement from the niche) and replacement. For this process, it is straightforward to determine the predicted clone size distribution as a function of the loss/replacement rate, λ, and the effective stem cell number in the crypt, N (10 and supplementary theory).

Our observations suggest that K-ras activation in stem cells confers a survival advantage over their WT neighbors. However, the bias appears to be limited, since the chance of ‘extinction’ for K-ras clones is still present albeit diminished. To accommodate this bias, we supposed that a K-ras mutant clone can expand by stochastic stem cell division displacing a WT stem cell neighbor at a rate λ(1+δ), while the reverse process (duplication of a WT stem cell leading to displacement and loss of K-ras mutant stem cell neighbor) occurs at a rate λ(1-δ). The parameter δ controls the size of the imbalance (supplementary theory). In WT, any one of the stem cells in a crypt can give rise to crypt colonization with equal probability. By contrast, following K-ras activation, the K-ras mutant clone has a higher probability of colonizing a crypt than its WT neighbors.

To apply the neutral and biased drift model to the WT and K-ras data, we must first define the effective stem cell number, N. Recent studies based on multi-day imaging of lineage traced clones that are initiated in Lgr5 intestinal stem cells, suggest that stem cells positioned near the boundary of the niche are temporally biased towards displacement from the niche and loss of stemness, suggesting the effective stem cell number is less than the number of Lgr5^hi^ cells (L. Ritsma, S.I.J. Ellenbroek, J. Van Rheenen, personal communication). These findings are corroborated by a recent lineage tracing study by Kozar *et al* 22 that defines a small ‘functional’ stem cell population. Here, based on the quantitative analysis of Ritsma *et al*, we take a figure of N = 8, noting that, while we are concerned with the relative difference between WT and K-ras mutant cells, our conclusions are largely insensitive to the precise choice.

**Figure 2:**
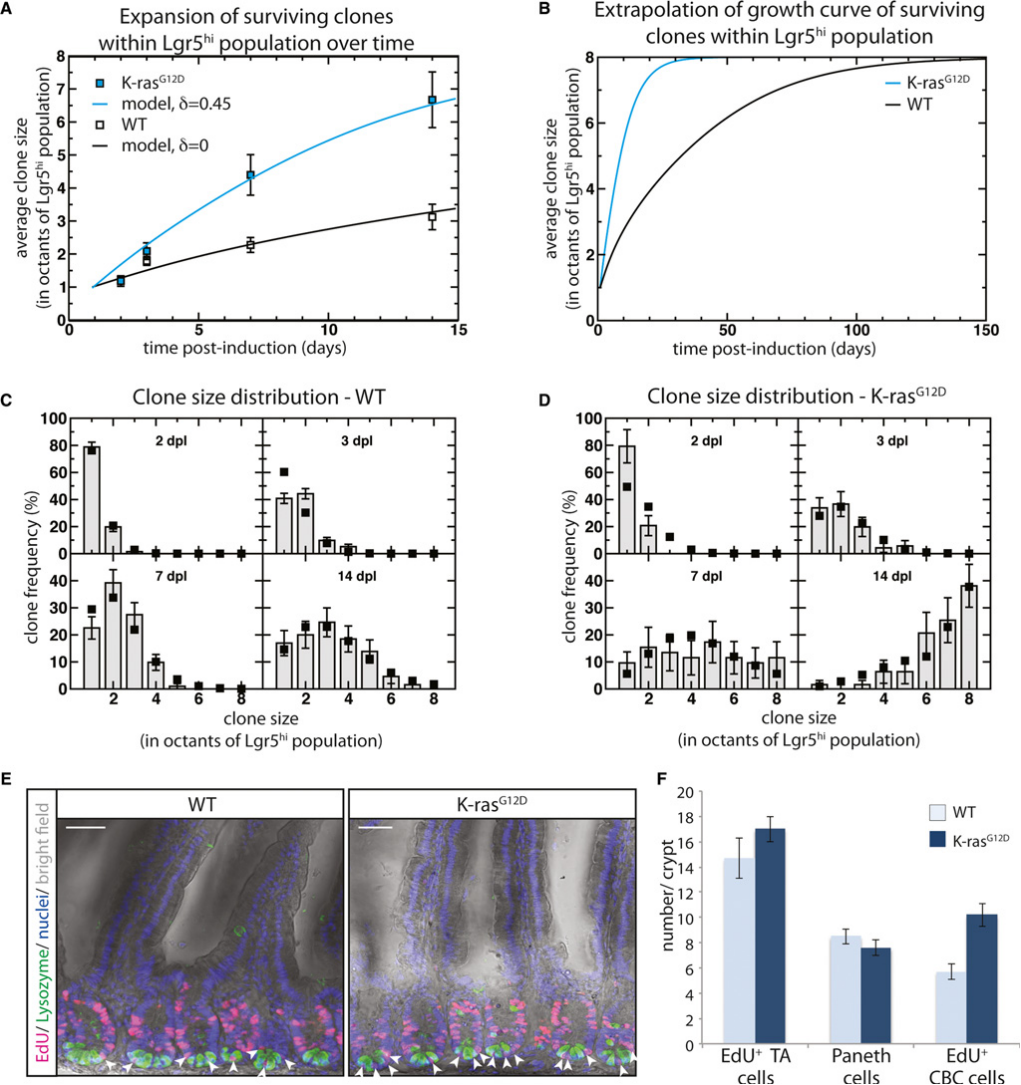
K-ras^G12D^ mutated Lgr5^hi^ cells follow a pattern of biased drift due to a faster cell cycle Average size of K-ras clones within the Lgr5^hi^ stem cell compartment over time, with WT data shown for comparison. Open and blue squares show experimental data from the K-ras mutant and WT clones, respectively, at day 2, 3, 7 and 14 post-induction (data are represented as mean ± SEM.) Black and blue lines show a modeled fit of the biased drift dynamics to the data. The parameter δ controls the degree of imbalance; with δ = 0.45±0.05 (biased) δ = 0 (unbiased) (supplementary theory). In both cases, we separated the crypt in octants, translating the effective stem cell number, *n* = 8.Extrapolation of the growth curves from (A) over a longer period of time showing the speed with which the biased drift dynamics converges towards monoclonality of the crypt.Clone size distribution of the marked clones in the WT background shows an excellent fit of the experimental data (bars) to the neutral drift model (squares) in (A).Clone size distribution of the K-ras mutant clones shows an excellent fit of the experimental data (bars) to the biased drift model when the bias is set to δ = 0.45 (squares).Confocal image of proximal small intestinal epithelium; either WT or K-ras^G12D^ activated, illustrate similar EdU (red) incorporation and no abnormal morphology after activation of K-ras^G12D^. Paneth cells are stained for lysozyme (green). Arrows point to EdU^+^ intestinal CBC stem cells. Scale bars; 50 μm.Quantification of EdU^+^ CBC stem cells and Paneth cells in whole crypts, and EdU^+^ TA cells per cross-section, in WT and K-ras^G12D^ mice. More CBC stem cells per crypt enter the S-phase when mutant for K-ras^G12D^, indicating a faster cell cycle (> 100 crypts; 3 mice per group). Data information: In (A), (C), (D), error bars denote SEM and in (F), error bars denote STDEV.

Then, following a fit of the model dynamics to the average clone size using the clonal fate data from the study of the WT crypts 7, we find an overall stem cell loss/replacement rate of λ = 0.25 ± 0.05 per day (Fig2A and B, supplementary theory). With this rate, we find that the corresponding size distribution of clones agrees well with the model prediction (Fig2C). Then, using the same stem cell loss/replacement rate to define the competition of WT cells with their K-ras neighbors, *viz*. λ(1-δ) = 0.25 per day, a fit to the average clone size of K-ras mutant clones gives a bias of δ = 0.45 ± 0.05 (Fig2A and B, supplementary theory). Once again, a comparison of the model prediction with the observed clonal fate data provides good agreement (Fig2D). With such a bias, the survival probability of a K-ras mutant cell in a WT background is around 65%, a factor of 4–5 larger than a WT cell in a WT background. Indeed, given that Cre-recombination efficiency shows a degree of variability between genetic loci, it is likely that this analysis provides a small underestimate of the true scale of bias (supplementary information).

Considering the hyperplasia effects of oncogenic K-ras in tissues other than small intestine 14 15 16 17, it is likely that the bias in survival potential in K-ras mutant stem cells finds its origin in an increased cell division rate. To check the cell cycle status of intestinal stem cells, a pulse of EdU was administered to assess the frequency of CBC stem cells in WT and K-ras^G12D^ epithelia that enter the S-phase. Indeed, more EdU^+^ CBC stem cells were obtained per crypt in K-ras mutant epithelia, while the number of Paneth cells, crypt size and the overall morphology of the intestine looked normal (Fig2E and F), indicating that their cell cycle rate is faster. As intestinal stem cells are reported to undergo approximately one cell cycle per day 23, an increase of one cell cycle per 15 h, a number close to the 12–16 h cell cycle time reported for the transit amplifying progenitors 24, would already be sufficient to produce the phenotypic effects as observed in our experiments. This increased cell cycle rate explains, at least in large part, the underlying mechanism by which K-ras mutant stem cells acquire a competitive survival over WT stem cells.

### Clonal expansion of mutant K-ras via crypt fission

In neonatal animals and humans, the intestinal epithelium grows through the duplication of crypts by the process of crypt fission. Although the rate of crypt fission in steady state epithelium is low, the frequency can increase as a regenerative response to epithelial injury or due to mutations 25 26 27 28. Yet, most studies have not been able to directly visualize crypt ancestry.

To determine the impact of an oncogenic *K-ras* mutation on crypt fission in adult mouse intestine, we induced sporadic lineage tracing in Lgr5 stem cells containing *R26R-Confetti*. Over time, a certain amount of Confetti-marked Lgr5 stem cells will successfully colonize the entire crypt as the stochastic outcome of neutral (in the case of WT) or biased (for K-ras) drift dynamics. When such a marked crypt undergoes crypt fission, the duplicated crypt will bear the same fluorescent mark.

**Figure 3:**
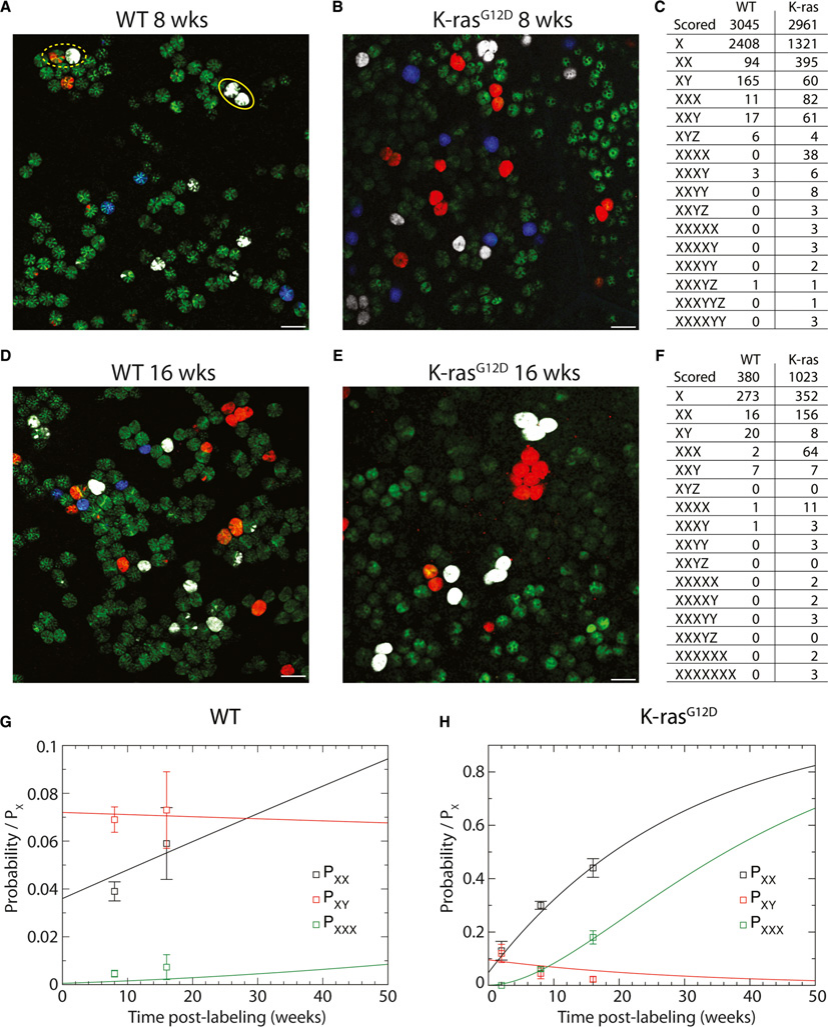
K-ras^G12D^ mutant clones expand through enhanced crypt fission A  Small intestinal crypts with sporadically activated Lgr5^hi^ stem cells. Lgr5 stem cells are in green. R26R-Confetti clones are visualized and marked with YFP (pseudo color white), RFP (red) or membrane tagged CFP (blue). After 8 weeks, the majority of surviving WT clones dominated their crypt as the outcome of neutral drift dynamics. Adjacent labeled crypts either had the same color ‘XX’ (ellipse), or different colors ‘XY’ (dashed ellipse).B  As in (A) but for K-ras mutant clones.C  Quantification of labeled crypt clusters for WT and K-ras mice 8 weeks after labeling. The number of XX crypt pairs is significantly higher than in the WT situation.D–F  As in (A–C) but after 16 weeks of tracing. Patches of multiple adjacent labeled crypts appeared. Patches were larger and more frequent in K-ras mutant crypts.G  Probability of obtaining two neighboring monoclonal crypts of the same (P_XX_) or different (P_XY_) color in WT normalized by the chance that an isolated crypt is fully labeled, P_x_. P_XXX_ represents the probability to finding a cluster of three crypts with the same color, normalized by the probability P_x_. Points represent experimental data derived from B, and the lines represent the modeled fit of the crypt fission dynamics with induction frequency *p*_*0*_^WT ^= 0.006 ± 0.001 per crypt and a crypt fission rate of *f*^WT^=0.01±0.002 per 8 weeks per crypt (main text and supplementary theory).H  As in (G), but for K-ras mutant clones. In this case, a fit of the data leads to an induction frequency of *p*_*0*_^Kras ^= 0.004 ± 0.001 per crypt and a crypt fission rate of *f*^K^^ras^=0.3±0.04 per 8 weeks per crypt. Data information: In (G) and (H), error bars denote SEM. Scale bars, 100 μm.

After 8 weeks of tracing, we scored ‘labeled’ crypts (defined as crypts in which more than 50% of the stem cell compartment is labeled by a single color) involving any of three visible *R26R-Confetti* colors (Fig3). Adjacent labeled crypts with the same fluorescent marking were scored as ‘XX’ (ellipse). Crypt pairs with different colors were scored as ‘XY’ (dashed ellipse). Note that ‘XX’ crypt pairs can result either from crypt fission or from chance labeling of stem cells in neighboring crypts, whereas ‘XY’ crypt pairs can only occur by chance. The ratio of XX versus XY crypt pairs was significantly higher in K-ras mice compared to WTs, indicating more crypt fission events in K-ras mice (Fig3A–C).

After 16 weeks of tracing, the number of crypt fission events in WT mice had increased slightly. Crypt fission events did not occur in a uniform fashion. Certain patches of epithelia had significantly more crypt fission events than could be expected by the average number of fissions. Some crypts probably duplicated multiple times (Fig3D, cluster of four red crypts at top of panel). In K-ras mice, large patches of monochromatic crypts were observed after 16 weeks of tracing (Fig3E–F). These patches contained up to eight crypts, implying a significantly higher rate of crypt fission. Of note, in WT mice, patches of more than four crypts were not observed after 16 weeks or even 30 weeks of tracing.

### Quantitative analysis of enhanced crypt fission rate

To address the significance of the crypt fission data and to infer the fission rate in WT and K-ras mice, we turned to a more quantitative analysis. By focusing on the 8 and 16 week timepoints to analyze the fission data, we could neglect details of the internal composition of labeled crypts since the majority of clones that survived up to that time already colonized the entire crypt (supplementary theory). In addition, the recombination efficiency was approximately equal for the three different colors. Therefore, the relative contribution of independent recombination events versus crypt fission could be enumerated (supplementary theory). To estimate the contribution from crypt fission, the chance of a monoclonal crypt undergoing *n* rounds of fission can be modeled by a birth-type process (supplementary theory). In analyzing the data, we determined that the impact of crypt death is minimal (supplementary theory).

By comparing the frequencies of different crypt clusters (FigC and F), we found the induction frequency (resulting in monoclonal crypts) for WT mice was set by *p*_*0*_^WT^ = 0.006 ± 0.002 per crypt. By fitting the scored clusters to the model, we found a crypt fission rate of *f*^WT^ = 0.01 ± 0.002 per 8 weeks per crypt. This figure confirms that crypt fission in steady state epithelium in adult mice is indeed rare. For the K-ras mice, the clonal induction frequency was comparable to WT mice at *p*_*0*_^Kras^ = 0.008 ± 0.002 per crypt. However, the crypt fission rate of *f*^Kras^ = 0.27 ± 0.04 per 8 weeks per crypt, was a factor of 30 higher. As well as providing an excellent prediction for the 2 and 16 week timepoints, these figures also correctly predicted the fraction of 3-crypt clusters, XXX (Fig3G and H) as well as higher order clusters (supplementary theory). In this case, crypt fission following K-ras activation was predicted to generate an exponential distribution of cluster sizes.

**Figure 4:**
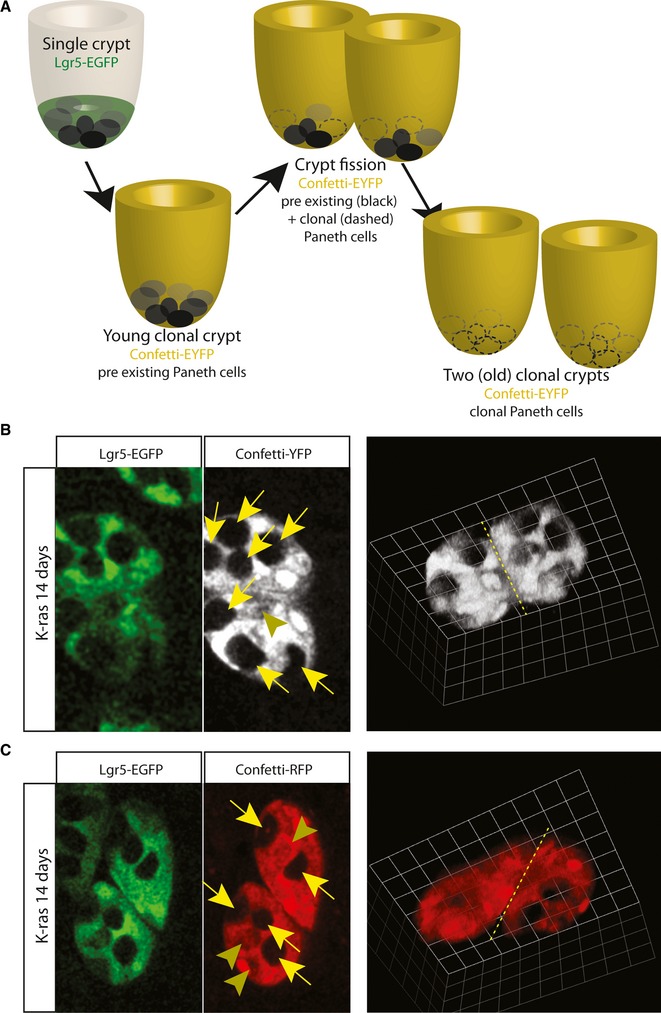
Existing Paneth cell niche is divided over both new crypts Schematic representation of experimental setting. Due to fast colonization and enhanced crypt fission rate of K-ras mutant cells, we captured crypts undergoing fission (physical connected between two halves higher up in the crypt) that contained Paneth cells that already existed prior to clonal marking (non-labeled). Distribution of pre-existing Paneth cells over both halves suggests that fission involved splitting of the existing stem cell population and its niche, rather than single stem cell derived clones that branches off the existing crypt.Left panels, bottom view of crypt undergoing fission. Lgr5^hi^ cells (green) are all clonally marked by Confetti-YFP (pseudo color white). Pre-existing Paneth cells, not marked by Confetti-YFP, are divided over both new crypts (yellow arrows). Arrowhead points to newly derived clonal Paneth cell. Right panel, 3D rendering of the crypt fission to illustrate the connection still present between the two young crypts (yellow dashed line).Results using the experimental setting outlined in (A), but the clone is marked by Confetti-RFP (red) rather than Confetti-YFP.

Crypt fission or branching has been known to occur during growth, repair and homeostasis in mice as well as in humans 29 30. Crypt size tends to correlate with fission rate and the bifurcation plane is randomly orientated with respect to the villus base 25. However, little is known how crypts bifurcate. Our experimental setting allowed a unique opportunity to provide mechanistic insight (Fig 4A). Due to the fast crypt colonization of K-ras mutated stem cells, we were able to score clonal crypts after 14 days of tracing that still contained Paneth cells that already existed prior to the clonal labeling. These Paneth cells were identifiable by the absence of clonal Confetti labeling. Due to the enhanced crypt fission rate of K-ras mutated crypts, a sufficient number of these clonal crypts underwent fission. In all 12 events that we scored as crypt fission after 14 days of tracing, we noticed distribution of pre-existing Paneth cells to both sides of the branching crypt buds (Fig4B and C), suggesting that crypt fission doesn't involve a single stem cell derived bud that branches off the parental crypt. Future studies are needed to reveal how many stem cells per bud are exactly required to establish a stable bifurcation that ultimately transforms into two independent crypts.

Although we assumed that the timing between consecutive divisions is statistically uncorrelated, the exact mechanistic process that promotes crypt fission, such as an overload of stem cells and Paneth cells, is unknown 25. In our hands, as well as previously documented, the Lgr5 stem cell pool, as well as the average crypt size, remains roughly unaltered after mutating K-ras 31. Alternatively, fission as a response to tissue injury might correlate with the non-uniform spreading of crypt fission. Analysis of human samples indeed suggests that fission is more common in crypts isolated from adenomas and hyperplastic polyps 32.

During the last couple of years, more and more tissues reveal homeostatic mechanisms that rely on neutral competition between stem cells. Our results can therefore be considered in a much wider perspective, as it offers new insights into how neutral drift phenomena can be subverted by mutations to expand in fields of mutant cells while not creating phenotypic abnormalities. The expansion and spread of mutations that do not directly lead to morphological abnormalities can nevertheless dramatically increase the ‘target size’ for additional oncogenic mutations that may lead to tumor formation. For instance, in a normal intestine, only the stem cell population is reported to be the cell-of-origin for adenoma formation 11. However, virtually all the cells, including villus cells, represent potential cell-of-origins for progressive adenoma development in an epithelium with oncogenic *K-ras* mutations 33.

Clinical observations, such as polyclonal human colorectal adenomas, suggest a pre-cancerous field defect that facilitated independent adenoma formations in close proximity of each other 34. Indeed, similar genetic and epigenetic profiles are observed in multiple colorectal cancers from individual patients 35. The ability to identify pre-tumorigenic cells and to understand and manipulate their behavior may prove to be important for future therapies.

## Materials and Methods

### Mice

Lgr5-EGFP-Ires-CreERT2 mice were bred with K-rasLSL-G12D and R26R-Confetti mice. Triple heterozygous mice of 10 weeks of age were used for experiments (referred to as K-ras mice). For WT experiments, Lgr5-EGFP-Ires-CreERT2/R26R-Confetti mice were used. Lgr5 cells are marked with green fluorescent protein EGFP and express a tamoxifen-inducible version of Cre that can be used to activate *K-ras*^*G12D*^ and *R26R-Confetti* alleles. R26R-Confetti is a multicolor Cre-reporter that expresses one out of four possible fluorescent proteins (nuclear Green, Yellow, Red or membrane tagged Blue) as a random outcome of the recombination process. Tissue preparation and analysis by confocal microscopy was performed as described (supplementary information) 7 23.

Cre-recombination efficiency shows a degree of variability per genetic locus. Therefore, clones exist that express oncogenic K-ras but that are not marked with Confetti (false negatives, i.e. 169 clones/2000 crypts) and vice versa (false positives, i.e. 8 clones/ 2000 crypts, versus 383 true positives/2000 crypts). Both scenarios in fact skew the data towards an underestimation of true scale of the bias.

### EdU incorporation

K-rasLSL-G12D mice were bred with villinCreERT2 mice. Double heterozygous mice of 10 weeks of age were used for experiments. For WT controls, only villinCreERT2 heterozygous littermates were used. Around 4 days after induction with 5 mg tamoxifen, EdU (100 μl 10 mM) was injected 2 h prior to sacrifice. Crypts were scanned using Leica Sp8X microscope, EdU^+^ and lysozyme^+^ cells were scored in 3D over >100 crypts of 3 mice per group. EdU^+^ TA cells were scored per cross-section over > 100 crypts of 3 mice per group. EdU^+^ cells adjacent to Paneth cells at the crypt base were scored as EdU^+^ CBC cells. EdU detection and lysozyme staining to mark Paneth cells was performed and analyzed as described 23.

## Supporting Information

Supporting Information

Review Process File
