# Supplementary material for: Biased competition between Lgr5 intestinal stem cells driven by oncogenic mutation induces clonal expansion
Source: EMBO Rep. 2013 Dec 16;15(1):62–9. doi: 10.1002/embr.201337799 (PMC3983678; doi:10.1002/embr.201337799)
Supplement: Supplementary file 1 [file embr0015-0062-sd1.pdf]

Supplementary figure 1
Clone dynamics during neutral competition between intestinal stem cells

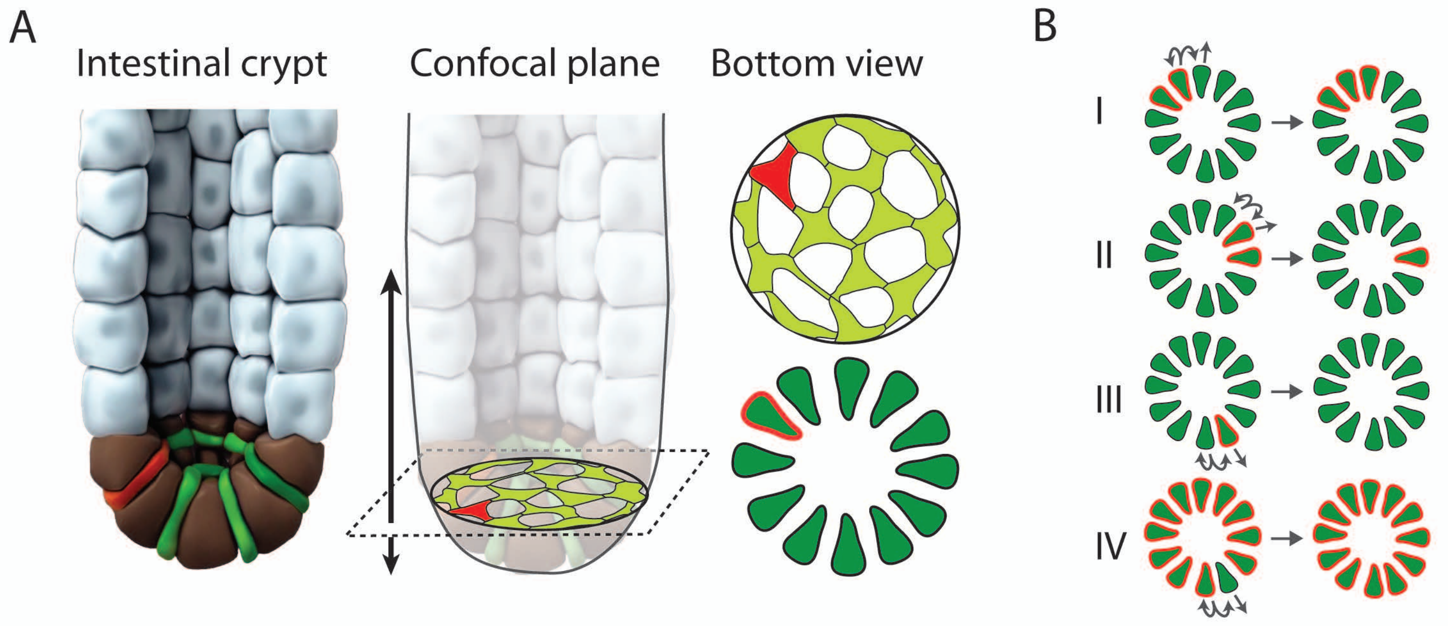

Supplementary figure 1: Clone dynamics during neutral competition between intestinal stem cells
A) Representation of an intestinal crypt. Lgr5 stem cells are represented in green, Paneth cells in brown and a single R26R-Confetti labelled stem cell is represented in red. Right panel: Lgr5 stem cell population in an intestinal crypt viewed from the bottom by confocal microscopy. The clones are analysed at multiple depths (dashed square) in the z-direction (arrows), starting form the bottom of the crypt towards the top. Clones can be represented for simplicity as a one-dimensional ring of cells that captures the essential aspects of neutral drift dynamics. Lgr5 stem cells are represented in green, Lgr5 stem cell that represents the mutant clone has red circumference. **B**) Division of stem cells can result in expansion (I) and contraction (II) of a labelled stem cell clone within the stem cell pool, ultimately resulting in loss (III) or fixation (IV) (labelled stem cells clones represented with red circumference).
