# Supplementary material for: Biased competition between Lgr5 intestinal stem cells driven by oncogenic mutation induces clonal expansion
Source: EMBO Rep. 2013 Dec 16;15(1):62–9. doi: 10.1002/embr.201337799 (PMC3983678; doi:10.1002/embr.201337799)
Supplement: Supplementary file 2 [file embr0015-0062-sd2.pdf]

# Supplementary figure 2

## Short-term clonal expansion of K-ras$^{G12D}$ mutated Lgr5$^{hi}$ cells

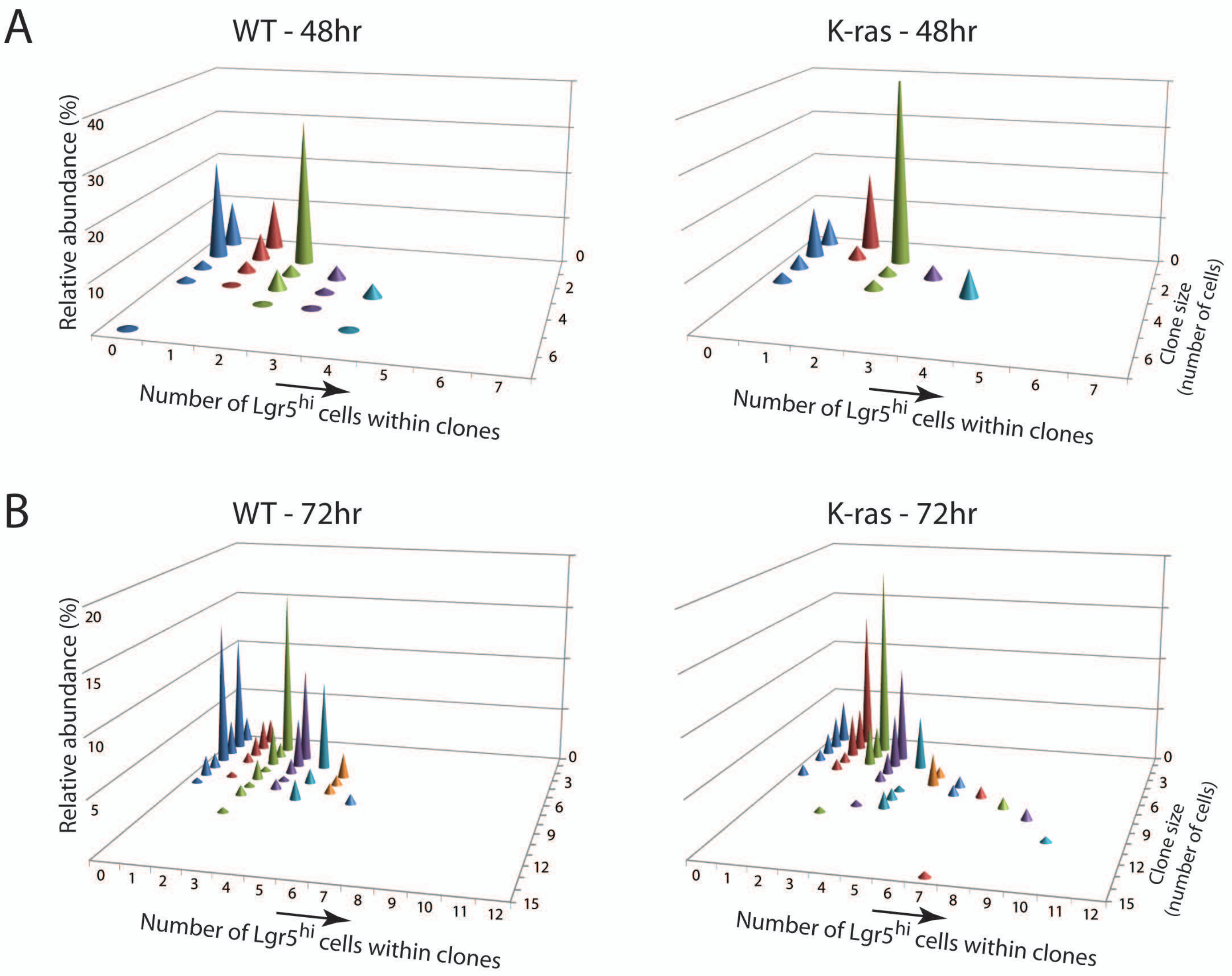


**Supplementary figure 2: Short-term clonal expansion of K-ras$^{G12D}$ mutated Lgr5$^{hi}$ cells**
**A**) Representation of the relative abundance (%) of clones scored for total clone size and the number of Lgr5$^{hi}$ cells after 48hr. Total clone size was scored as the total number of cells within a given clone, while the stem cell content was determined as the number of Lgr5$^{hi}$ cells within that clone. Left: WT clones, right: K-ras$^{G12D}$ activated clones. **B**) Like A, but after 72hrs of tracing.
