# Supplementary material for: Biased competition between Lgr5 intestinal stem cells driven by oncogenic mutation induces clonal expansion
Source: EMBO Rep. 2013 Dec 16;15(1):62–9. doi: 10.1002/embr.201337799 (PMC3983678; doi:10.1002/embr.201337799)
Supplement: Supplementary file 3 [file embr0015-0062-sd3.pdf]

SUPPLEMENTARY INFORMATION

Supplementary materials and methods

Mice

Lgr5-EGFP-Ires-CreERT2 mice (MGI:3764660) were bred with *K-rasLSL-G12D* (MGI:2429948) and *R26R-Confetti* mice (MGI:4835542). Triple heterozygous mice of 10 weeks of age were used for experiments (referred to as K-ras mice). For WT experiments, *Lgr5-EGFP-Ires-CreERT2/R26R-Confetti* mice were used. Lgr5 cells are marked with green fluorescent protein EGFP and express a tamoxifen-inducible version of Cre that can be used to activate *K-ras*^{G12D} and *R26R-Confetti* alleles. R26R-Confetti is a multicolor Cre-reporter that expresses one out of four possible fluorescent proteins (nuclear Green, Yellow, Red or membrane tagged Blue) as a random outcome of the recombination process [1]. Mice were mixed 129/Ola and Bl6 background. Raw data from WT short-term clonal tracing experiments was reproduced from Snippert et al., 2010. To rule out effects due to strain differences, we repeated the experiment in WT littermate intestines (72 hours). The new data correlated well with the previously reported clonal fate data. Mice were induced with 5mg tamoxifen. Recombination of *Confetti* to EGFP is inefficient, while the three other colors appear with equal frequency. For each time point, at least two mice were analyzed and scored.

Cre-recombination efficiency shows a degree of variability per genetic locus. Therefore, clones exist that express oncogenic K-ras but that are not marked with Confetti (false negatives, i.e. 169 clones/ 2000 crypts) and vice versa (false positives, i.e. 8 clones/ 2000 crypts, versus 383 true positives/ 2000 crypts). Both scenarios in fact skew the data towards an underestimation of true scale of the bias. For scoring false negatives and false positives, consecutive sections of intestines after 1 week of tracing were stained against pERK (active K-ras) or GFP recognizing all fluorescent protein variants. The number of clones reaching the TA/villus compartment was scored in two different mice.

EdU incorporation

K-rasLSL-G12D mice were bred with villinCreERT2 mice. Double heterozygous mice of 10 weeks of age were used for experiments. For WT experiments, only villinCreERT2 heterozygous littermates were used. Mice were induced with 5mg tamoxifen. Around 4 days, EdU (100ul 10mM) was injected 2 hours prior to sacrifice. EdU detection and lysozyme staining to mark Paneth cells was performed as described [2]. Crypts were scanned using Leica Sp8X microscope, EdU and lysozyme^{+} cells were scored in 3D over > 100 crypts of 3 mice per group. EdU^{+} TA cells were scored per cross-section over > 100 crypts of 3 mice per group. EdU^{+} cells adjacent to Paneth cells at the crypt base were scored as EdU^{+} CBC cells.

Tissue preparation and analysis by confocal microscopy

For whole-mount imaging, intestines were opened along their length and villi were scraped off using a microscope glass. Subsequently proximal small intestines were cut in parts, fixed in 4% Paraformaldehyde at room temperature for 30 minutes and washed in cold PBS. Next, parts were prepared free from connective tissue and muscle layers, transferred to a new

microscope slide with crypt bottoms oriented to the top, embedded with vectashield (Vector Laboratories) and sealed with coverslips. Confocal images were acquired using a Leica Sp5 AOBS microscope. Images were processed using ImageJ, Photoshop and Volocity (PerkinElmer). Threshold for $Lgr5^{hi}$ cells was set at 66% of the average signal from 10 bright GFP positive cells at the entire base of the crypt (as previously described [1]). Crypts harboring multiple clones upon induction were excluded from the analysis since these clones will compete with each other on a neutral basis.

K-ras induced phenotypes in the mouse

Our findings of minor morphological abnormalities in the small intestine are similar to data from previous reports [3-5] where in each case inducible versions of Cre were used. However, these findings contrast with data from transgenic overexpression of mutant K-ras [6] or activation of endogenous $K\text{-}ras^{G12D}$ at early stages in development via villinCre, Fabpl-Cre or CDX2-G22Cre [7-9].

This may be due to the timing of $K\text{-}ras^{G12D}$ activation. For instance in pancreas, embryonic activation results in neoplasias with occasional advanced lesions [10]. Activation in adult acinar cells [11, 12], or adult ducts [5, 13] rarely yield neoplasias during normal homeostasis. A similar phenomenon may occur in the small intestine, in which the effects of $Kras^{G12D}$ may vary per developmental stage. Subtle differences in mouse genetic backgrounds may play a role as well, since differences have been observed between research groups that used the same villinCre transgene [7, 14]. Alternatively, the exact location within the intestinal tract that is subject to the study may explain differential outcome of experiments. For instance, in our manuscript we focused mainly on the duodenum and jejunum parts, while Feng et al., used the same Lgr5-EGFP-ires-CreERT2 strain but specifically focused on colon and distal small intestine (because the other cre transgene they rely on, CDX2-G22Cre, only becomes activated during development in those parts of the gut) [9].

References:

1. Snippert HJ *et al* (2010) Intestinal crypt homeostasis results from neutral competition between symmetrically dividing Lgr5 stem cells. *Cell* **143:** 134-144
2. Schepers AG, Vries R, van den Born M, van de Wetering M, Clevers H (2011) Lgr5 intestinal stem cells have high telomerase activity and randomly segregate their chromosomes. *EMBO J* **30:** 1104-1109
3. Sansom OJ *et al* (2006) Loss of Apc allows phenotypic manifestation of the transforming properties of an endogenous K-ras oncogene in vivo. *Proc Natl Acad Sci U S A* **103:** 14122-14127
4. Luo F, Brooks DG, Ye H, Hamoudi R, Poulogiannis G, Patek CE, Winton DJ, Arends MJ (2007) Conditional expression of mutated K-ras accelerates intestinal tumorigenesis in Msh2-deficient mice. *Oncogene* **26:** 4415-4427
5. Ray KC, Bell KM, Yan J, Gu G, Chung CH, Washington MK, Means AL (2011) Epithelial tissues have varying degrees of susceptibility to Kras(G12D)-initiated tumorigenesis in a mouse model. *PLoS One* **6:** e16786
6. Janssen KP, el-Marjou F, Pinto D, Sastre X, Rouillard D, Fouquet C, Soussi T, Louvard D, Robine S (2002) Targeted expression of oncogenic K-ras in intestinal epithelium causes spontaneous tumorigenesis in mice. *Gastroenterology* **123:** 492-504
7. Trobridge P *et al* (2009) TGF-beta receptor inactivation and mutant Kras induce intestinal neoplasms in mice via a beta-catenin-independent pathway. *Gastroenterology* **136:** 1680-1688 e1687
8. Haigis KM *et al* (2008) Differential effects of oncogenic K-Ras and N-Ras on proliferation, differentiation and tumor progression in the colon. *Nat Genet* **40:** 600-608

9. Feng Y *et al* (2011) Mutant KRAS promotes hyperplasia and alters differentiation in the colon epithelium but does not expand the presumptive stem cell pool. *Gastroenterology* **141:** 1003-1013 e1001-1010
10. Hingorani SR *et al* (2003) Preinvasive and invasive ductal pancreatic cancer and its early detection in the mouse. *Cancer Cell* **4:** 437-450
11. De La OJ, Emerson LL, Goodman JL, Froebe SC, Illum BE, Curtis AB, Murtaugh LC (2008) Notch and Kras reprogram pancreatic acinar cells to ductal intraepithelial neoplasia. *Proc Natl Acad Sci U S A* **105:** 18907-18912
12. Habbe N *et al* (2008) Spontaneous induction of murine pancreatic intraepithelial neoplasia (mPanIN) by acinar cell targeting of oncogenic Kras in adult mice. *Proc Natl Acad Sci U S A* **105:** 18913-18918
13. Guerra C, Schuhmacher AJ, Canamero M, Grippo PJ, Verdaguer L, Perez-Gallego L, Dubus P, Sandgren EP, Barbacid M (2007) Chronic pancreatitis is essential for induction of pancreatic ductal adenocarcinoma by K-Ras oncogenes in adult mice. *Cancer Cell* **11:** 291-302
14. Bennecke M, Kriegl L, Bajbouj M, Retzlaff K, Robine S, Jung A, Arkan MC, Kirchner T, Greten FR (2010) Ink4a/Arf and oncogene-induced senescence prevent tumor progression during alternative colorectal tumorigenesis. *Cancer Cell* **18:** 135-146

Supplementary Theory

In the main text, we discussed a biophysical modeling scheme to address both clonal evolution of stem cells within crypts following the activation of K-ras, and the dynamics of crypt fission. In the following sections, we provide a more detailed account of the theoretical schemes, leaving the central results for the main text.

1 Biased drift of the K-ras mutant stem cell population

1.1 Neutral drift in normal homeostasis

Under conditions of normal homeostasis, it has been shown that stem cells at the base of the crypt undergo a process of neutral competition in which the loss of a stem cell through commitment to differentiation is compensated by symmetrical self-renewal of neighboring stem cells [1,2]. As a result, single stem cell-derived clones undergo a pattern of neutral drift in which continual clonal loss is compensated by the expansion of neighboring clones (Suppl. Fig. 1). This process continues until all of the stem cells in a crypt become clonal.

To model clonal evolution on this background, following Refs. [1,2], we introduced a simple model in the main text that captures the fundamental aspects of the dynamics. In this model, the stem cell compartment is represented as a one-dimensional chain of equipotent cells which extend around the circumference of the crypt base region. Following stem cell division, neighbouring cells are displaced from the chain and commit to differentiation and loss. Through this process of stem cell loss and replacement, marked stem clones undergo neutral drift dynamics around the crypt base until the clone is lost or it expands across the entire chain and the crypt becomes monoclonal (i.e., fixed). The one-dimensional dynamics follows from the experimental observation that clones do not expand through the apex of the

crypt at the base. In this model, the resulting clone dynamics can be resolved analytically and depends on just two parameters – the effective loss/replacement rate, λ, of stem cells from the base of the crypt, and the total number of stem cells in the crypt, N. Note that, within this scheme, stem cell divisions leading to asymmetric fate do not change clone size. The loss/replacement rate is therefore distinct from the stem cell division rate.

Before summarising properties of the neutral clone dynamics, it is helpful to comment on the integrity of the model. Of course the one-dimensional arrangement of equipotent cells represents a caricature of the true stem cell organisation at the crypt base. Indeed, recent studies based on *in vivo* live-imaging using intravital microscopy suggest that the short-term self-renewal potential varies through the crypt base region, with stem cells at the border of the niche temporarily biased or "primed" towards displacement and differentiation, while those at the base region are biased towards survival (Ritsma et al., private communication). These studies show that, following division, crypt base progenitors can move interchangeabily between the border and base region, suggesting that Lgr5+ cells form a single, heterogeneous, stem cell pool. Fortunately, the dynamics of such a "multicomponent" system converge rapidly onto the effective one-dimensional model (on timescales comparable with the "equilibration" time – the typical time scale for transfer between border and base regions).

However, in making use of the effective one-dimensional model, we must therefore be careful to fix the total stem cell number, N, not by the number of Lgr5+ cells at the crypt base, but by an effective stem cell number which accounts for priming. From the results of the intravital live-imaging, this number is found to be approximately one half of the Lgr5+ population, consistent with the bias of cells at the base region towards self-renewal. Indeed, further evidence in support of this conjecture can be found in a recent and meticulous study of intestinal stem cell dynamics [3]. Following the results of the intravital live-imaging study of Ritsma et al, in this paper, we take the effective stem cell number to be around 8 in both the WT and K-ras mutant crypts. However, crucially, our conclusions rest, not on the precise number, but on the relative change of the loss/replacement rate following K-ras activation.

With this background, it is useful to summarise the results of the neutral dynamics of the one-dimensional model because (a) it will be used to analyze the clonal dynamics in the WT crypt and (b) it serves as a benchmark to describe the biased drift dynamics of cells following K-ras activation. In particular, at a time t following the genetic marking of a single stem cell,

one may show that the fraction of clones with $1 \leq n \leq N-1$ stem cells is given by [1],

$$P_n(t) = \frac{2}{N} \sum_{k=1}^{N-1} \sin\left[\frac{\pi k}{N}\right] \sin\left[\frac{\pi k n}{N}\right] e^{-4\sin^2\left[\frac{\pi k}{2N}\right]\lambda t}, \qquad 1 \leq n \leq N-1,$$

while the fraction of clones that have either become extinct, $P_0(t)$, or have saturated the crypt, $P_N(t)$, is given respectively by,

$$P_0(t) = \frac{2}{N} \sum_{k=1}^{N-1} \cos^2\left[\frac{\pi k}{2N}\right] \left(1 - e^{-4\sin^2\left[\frac{\pi k}{2N}\right]\lambda t}\right),$$
$$P_N(t) = \frac{2}{N} \sum_{k=1}^{N-1} (-1)^{k+1} \cos^2\left[\frac{\pi k}{2N}\right] \left(1 - e^{-4\sin^2\left[\frac{\pi k}{2N}\right]\lambda t}\right).$$

To apply these expressions, it is necessary to exclude crypts in which the number of labeled stem cells is zero, corresponding to clones that have (or will soon) become lost. We therefore define the 'persisting' clone size distribution as,

$$P_n^{(\text{pers.})} = \frac{P_n(t)}{1 - P_0(t)}, \qquad 1 \leq n \leq N.$$

At time scales in excess of the typical loss time $1/\lambda$, but shorter than the time scale for crypts to drift to monoclonality, N^2/λ, these equations enter a scaling regime where [1]

$$P_n^{(\text{pers.})} \approx \frac{1}{\langle n(t) \rangle} f(n/\langle n(t) \rangle),$$

with $\langle n(t) \rangle = \sqrt{\pi \lambda t}$ and $f(x) = \frac{\pi x}{2} \exp(-\pi x^2/4)$. With this background, we turn now to consider the dynamics of the K-ras activated cells in the field of WT cells.

1.2 Biased drift following K-ras activation

Following induction, it is evident from the clonal fate data (Fig. 1) that K-ras activation results in a strongly accelerated progression towards fixation. However, the extinction of a fraction of K-ras mutant clones at early times suggests that K-ras activation provides only a bias, with "drift" from larger to smaller clones still possible. To assess whether the dynamics of the mutant clones in the WT background can be described as a biased drift process, let us first consider the impact of such a bias on the simple one-dimensional model of the stem cell compartment. Specifically, let us suppose that, following the loss of a stem cell through

commitment to differentiation, a neighboring cell in which K-ras has been activated will have a higher chance of effecting its replacement through symmetrical cell division than a WT neighboring cell.

Let us, therefore, again define $P_n(t)$ as the probability of finding a K-ras mutant clone with $n \geq 0$ stem cells at a time t post-labeling. Then, if we define $\lambda(1+\delta)$ as the loss/replacement rate leading to expansion of the mutant clone, and $\lambda(1-\delta)$ as the rate leading to contraction, we obtain the Master equation for the time evolution of the probability,

$$\begin{aligned}\dot{P}_n(t) &= \lambda\hat{\Delta}P_n(t) - \lambda\left[(1-\delta)\delta_{n,1} + (1+\delta)\delta_{n,-1} - 2\delta_{n,0}\right]P_0(t) \\ &\quad -\lambda\left[(1-\delta)\delta_{n,N+1} + (1+\delta)\delta_{n,N-1} - 2\delta_{n,N}\right]P_n(t) + \delta_{n,1}\delta(t), \qquad (1)\end{aligned}$$

where, defining the one-dimensional lattice translation operator, $\hat{E}_m = e^{m\hat{k}}$ with $[\hat{k}, n]$=1, $\hat{\Delta} = \hat{\Delta}_0 - \delta(\hat{E}_1 - \hat{E}_{-1})$, and $\hat{\Delta}_0 = (\hat{E}_1 + \hat{E}_{-1}) - 2$ denotes the lattice Laplacian. The first term on the right-hand side of the equation describes the biased random walk of the boundaries of a labeled clone, the second term reflects the possibility of clone extinction, while the third term reflects the effect of clone fixation when all stem cells have become monoclonal. The final term imposes the initial boundary condition: the induction results in the labeling of just one stem cell per crypt at $t = 0$. Eq. (1) describes a discrete diffusion equation on the interval $1 \leq n \leq N-1$, with absorbing boundaries at $n = 0, N$ imposed by the second and third terms of the equation.

Before discussing the solution of Eq. (1), it is useful to reflect on the biological source of the bias. In principle, the bias of clonal fate towards expansion may reflect an "active" process in which K-ras mutant cells promote the loss of neighbouring WT cells. Equally, the bias may reflect the "passive" effect of a differential cell cycle time. If stem cell loss and replacement is a manifestation of symmetrical stem cell division promoting the displacement of neighbours form the niche, with attendant loss of stemness, cells which acquire a proliferative advantage will be equally biased towards "survival". If the net stem cell loss/replacement rate scales in proportion to the stem cell division rate, we may therefore consider the ratio $(1+\delta)/(1-\delta)$ as reflecting the relative increase in the cell division rate. Intriguingly, from the study of proliferation kinetics using EdU incorporation, we do indeed find that much, if not all of the bias can be associated with the acceleration in the cell division rate.

To solve Eq. (1), we note that for $1 \leq n \leq N-1$ it is sufficient to solve the discrete

diffusion equation,

$$\dot{P}_n(t) = \lambda \hat{\Delta} P_n(t)\,, \tag{2}$$

subject to the initial condition $P_n(0) = \delta_{n,1}$, and "artificial" boundary conditions $P_0(t) = P_N(t) = 0$. These boundary conditions do not reflect the true behaviour of $P_0(t), P_N(t)$, but they ensure that Eqs. (1) and (2) give the same results for $P_n(t)$ with $1 \leq n \leq N-1$. Once the solution for $1 \leq n \leq N-1$ is known, it is a simple matter to calculate $P_0(t)$ and $P_N(t)$ by integrating Eq. (1) to obtain,

$$P_0(t) = \lambda(1-\delta)\int_0^t P_1(t')dt'\,, \qquad \text{and} \qquad P_N(t) = \lambda(1+\delta)\int_0^t P_{N-1}(t')dt'. \tag{3}$$

In contrast to the pure diffusion process (described by $\delta = 0$), the operator $\hat{\Delta}$ is non-Hermitian. However, we can restore the Hermitian diffusion form by effecting a similarity transformation. Setting $P_n(t) = v^n G_n(t)$, one obtains

$$\dot{G}_n = \lambda\left[(1-\delta)vG_{n+1} + (1+\delta)v^{-1}G_{n-1} - 2G_n\right].$$

Then defining $v = \sqrt{\frac{1+\delta}{1-\delta}}$ and $\mu = \sqrt{1-\delta^2}$, one obtains

$$\dot{G}_n = \mu\lambda\left[\hat{\Delta} - 2\left(\frac{1}{\mu} - 1\right)\right]G_n.$$

Finally, applying the boundary conditions on G_n, and setting $f_k = 2(\frac{1}{\mu} - 1) + 4\sin^2[\frac{\pi k}{2N}]$, the solutions of Eq. (2) take the form,

$$P_n(t) = v^n \sum_{k=1}^{N-1} a_k \sin\left[\frac{\pi k n}{N}\right] e^{-\mu\lambda t f_k}.$$

To fix the coefficients, a_k, we must make use of the boundary condition, $P_n(0) = \delta_{n,1}$. Making use of the orthogonality condition,

$$\frac{N}{2}a_k = \sum_{n=1}^{N-1} v^{-n} P_n(0) \sin\left[\frac{\pi k n}{N}\right] = \frac{1}{v}\sin\left[\frac{\pi k}{N}\right],$$

we obtain

$$P_n(t) = \frac{2}{N} v^{n-1} \sum_{k=1}^{N-1} \sin\left[\frac{\pi k}{N}\right] \sin\left[\frac{\pi k n}{N}\right] e^{-\mu\lambda t f_k}.$$

We can now use this result to obtain the extinction and fixation probability. Making use of Eq. (3), we obtain

$$P_0(t) = \sqrt{\frac{1-\delta}{1+\delta}} \frac{2}{N} \sum_{k=1}^{N-1} \frac{1}{f_k} \sin^2\left[\frac{\pi k}{N}\right] \left[1 - e^{-\mu\lambda t f_k}\right]$$

$$P_N(t) = \left(\frac{1+\delta}{1-\delta}\right)^{(N-1)/2} \frac{2}{N} \sum_{k=1}^{N-1} \frac{(-1)^{k+1}}{f_k} \sin^2\left[\frac{\pi k}{N}\right] \left[1 - e^{-\mu\lambda t f_k}\right].$$

1.3 Analysis of the experimental data

With the theoretical background in place, we now turn to the analysis of the experimental data. As discussed above, to address the experimental data, we cannot fix the number of stem cells, N, by the number of Lgr5+ cells. Instead, following Ritsma *et al.*, we take the effective stem cell number, $N = 8$, noting that the qualitative conclusions will be largely insensitive to the precise value. To quantify the size of the clone from the experimental data, we take the fraction of fluorescently labelled cells in proportion to the total number of Lgr5+ cells at the crypt base (estimated at between 14 and 16 for the small intestine), divided into octants. For example, a clone with 1-2 marked cells out of 16 would belong to the first octant, 3-4, the second, and so on. Here, by choosing octants, we note that each effective stem cell contributes to 1/8 of the total crypt cells, on average.

To calibrate the method, and obtain an estimate for the effective loss/replacement, we can make use of the WT clonal fate data obtained in the study of Ref. [2]. By fitting the effective steady-state stem cell loss/replacement rate, to the observed increase in the average clone size, we obtain a figure of $\lambda = 0.25 \pm 0.05$/day (Fig. 2A,B). (Here we have included a time offset of around 24 hours to account for the delayed activity of the Cre following drug administration.) With this value, comparison of the clone size distributions with theory provides an excellent agreement of the model with experiment (Fig. 2C).

To analyze the dynamics of cells following Kras activation, we can follow the same procedure taking, once again, $N = 8$. In this case, we have to fit the average clone size to both the

average loss/replacement rate, λ, and the bias, δ. However, to further constrain the model, we will suppose that the largest contribution towards the bias drift derives from the substantial increase in the cell division time of the K-ras mutant cells, as evidenced by the proliferation assay using short-term EdU incorporation. In this case, we may set the rate at which Kras activated cells are lost and replaced by neighbouring WT cells by the loss/replacement rate in WT tissue, i.e. the bias conferred by Kras activation is a passive one leaving the dynamics of the WT neighbours unchanged. We therefore impose the constraint $\lambda(1-\delta) = 0.25$/day. Then, by fitting the model to the average clone size, we obtain a bias of $\delta = 0.45 \pm 0.05$ (Fig. 2A,B). Once again, with these parameters, comparison of the measured clone size distribution reveals an excellent fit of the model to the experimental data (Fig. 2D).

This completes our analysis of the clonal fate data. In the next section, we turn to consider the question of crypt fission.

2 Analysis of the crypt fission data

From the quantitative analysis of the short-term clonal fate data, we found that K-ras activation confers a major survival (and proliferative) advantage of the mutant intestinal stem cells over that of wild-type. However, by itself, this result does not disclose whether the frequency of crypt fission is perturbed following K-ras activation. In the following section, we will develop a theoretical approach to study the development of crypt fission events following genetic labeling of tissue.

2.1 Crypt fission: theory

To undertake this programme, let us begin by defining p_{X} as the probability that a crypt is both marked at induction by color X and becomes fixed over time (defined operationally as acquiring more than 50% labeled cells of color X). Since we find that the three confetti colors that label efficiently are approximately equally represented, we set $p_{\mathrm{X}} = p_0$ for all colors. (Note that we found no marking of the fourth color, the nuclear GFP.) Although we are interested in the problem of crypt fission, it is important to recognize that crypts of a common color can "cluster" by chance, i.e. stem cells of the same color X can, with some probability, be induced in neighboring crypts and both drift to monoclonality. To assess the crypt fission frequency, we must be careful to take into account this contribution to the

statistics.

Therefore, let us first consider the cluster statistics when crypts do not undergo fission at all. In this case, if we suppose that the induction frequency is low (i.e., the probability that the crypt becomes marked and drifts to monoclonality is very small, $p_0 \ll 1$), an assumption that will be tested self-consistently below, the chance that a labeled monoclonal crypt is found isolated, surrounded by unmarked near-neighbours, is given simply by

$$P_{\rm X} \simeq n_{\rm color} \times p_0 \, ,$$

where $n_{\rm color} = 3$ denotes the number of colors. (Here, we work with a general value for the number of colors, $n_{\rm color}$, so that the color dependence can be monitored below.) The chance that two neighbors have the same color is then given by

$$P_{\rm XX} = n_{\rm color} \times z p_0 (1 - p_0)^{z-1} \times p_0 \simeq n_{\rm color} \times z p_0^2 = z p_0 P_{\rm X} \, ,$$

where z denotes the average coordination of neighboring crypts. Empirically, this figure is found to be similar to the “close-packing density”, 6. Similarly, the chance of finding two neighboring monoclonal crypts of different colors is given by,

$$P_{\rm XY} \simeq n_{\rm color}(n_{\rm color} - 1) \times z p_0^2 = (n_{\rm color} - 1) P_{\rm XX} = (n_{\rm color} - 1) z p_0 P_{\rm X} \, .$$

Finally, turning to three crypt clusters (all neighbours), we find that

$$\begin{aligned} P_{\rm XXX} &\simeq n_{\rm color} \times \frac{1}{2} z(z-1) p_0^3 = \frac{1}{2}(z-1) p_0 P_{\rm XX} = \frac{1}{2} z(z-1) p_0^2 P_{\rm X} \\ P_{\rm XXY} &\simeq 3(n_{\rm color} - 1) P_{\rm XXX} \\ P_{\rm XYZ} &\simeq n_{\rm color}(n_{\rm color} - 1)(n_{\rm color} - 2) P_{\rm XXX} \, , \end{aligned}$$

and so on. Evidently, with each additional crypt, the probability of finding the cluster is suppressed by a factor p_0. Therefore, at low induction frequency, $p_0 \ll 1$, the chance induction of high cluster sizes becomes negligibly small. Together, these expressions represent the baseline probabilities of crypt clustering due to chance induction and fixation events in neighbouring crypts.

With these expressions in hand, let us now turn to consider the contribution to clustering of fixed crypts from the (infrequent) process of crypt fission. Later, we will consider the potential influence of crypt extinction on the statistics. If we assume that crypt fission follows

a Poisson random process in which the time between consecutive fission events is statistically uncorrelated (i.e. Markovian), with a crypt fission rate, f, the chance that a labeled crypt has expanded to generate a cluster of n labeled crypts is controlled by a simple birth-type process, $\mathrm{C} \mapsto \mathrm{C} + \mathrm{C}$, and given by

$$F_n(t) = e^{-ft}(1 - e^{-ft})^{n-1}, \qquad n > 0\,.$$

The result for F_1 is easy to understand: $F_1 = e^{-ft}$ simply denotes the decay in the survival probability of single isolated labeled crypts due to crypt fission. Moreover, at long times, $ft \gg 1$ (which may translate to an unreasonably long time for the experimental system), the cluster size distribution approaches the form of an exponential,

$$F_n(t) = \frac{1}{\langle n(t) \rangle} e^{-n/\langle n(t) \rangle}\,,$$

where $\langle n(t) \rangle = e^{ft}$ denotes the average cluster size, i.e. without compensation by crypt death and loss, the average crypt number would be predicted to rise exponentially, albeit with a potentially very small exponent.

Pieced together, these two types of contribution (sporadic chance induction of clusters, and the aggregation of marked crypts due to fission) lead to the first few results in the "hierarchy": For a crypt to remain as single and isolated, it must be created as such, and not undergo fission, viz.

$$P_{\mathrm{X}}(t) = n_{\mathrm{color}} p_0 F_1(t) = n_{\mathrm{color}} p_0 e^{-ft}$$

Similarly, for two neighbouring marked crypts to survive, they must be induced as such, and neither crypt must undergo fission, viz.

$$P_{\mathrm{XY}}(t) = n_{\mathrm{color}}(n_{\mathrm{color}} - 1) z p_0^2 F_1^2(t) = (n_{\mathrm{color}} - 1) z p_0 e^{-ft} P_{\mathrm{X}}(t)$$

By contrast, two neighbouring crypts of common color could derive either from chance induction as neighbours, or following the fission of a single isolated crypt, viz.

$$P_{\mathrm{XX}}(t) = n_{\mathrm{color}} z p_0^2 F_1^2(t) + n_{\mathrm{color}} p_0 F_2(t) = (z p_0 e^{-ft} + 1 - e^{-ft}) P_{\mathrm{X}}(t)\,.$$

For higher order clusters, the number of "permutations" that contribute increases rapidly. For example a cluster with three marked crypts, XXX, can be generated by chance, without any crypt fission. It could also arise from a chance pair followed a single fission event, or a

single crypt can undergo two rounds of fission, leading to the expression,

$$\begin{aligned} P_{\mathrm{XXX}}(t) &= n_{\mathrm{color}}\frac{1}{2}z(z-1)p_0^3F_1^3(t) + n_{\mathrm{color}}zp_0^2 \times 2F_2(t)F_1(t) + n_{\mathrm{color}}p_0F_3(t) \\ &= \left[\frac{1}{2}z(z-1)p_0^2e^{-2ft} + zp_0 2e^{-ft}(1-e^{-ft}) + (1-e^{-ft})^2\right] P_{\mathrm{X}}(t)\,. \end{aligned}$$

Although it is possible to enumerate straightforwardly higher order cluster contributions, such a program rapidly becomes unwieldy. Fortunately, for low induction frequencies, $p_0 \ll 1$, the leading contribution to the probability derives from fission events, with subleading terms suppressed by a factor of order $zp_0/(1-e^{-ft})$. Taking only this leading contribution, we have

$$P_{n\times X} = (1-e^{-ft})^{n-1}P_{\mathrm{X}}\,.$$

2.2 *Fit of the model to the data*

With these results in hand, we now turn to the comparison of the model with the measured experimental data. Starting with the WT system, the steady progression of crypts towards monoclonality implied by neutral drift does not allow a reliable assessment of the frequency of fixed crypts. At these early times, the vast majority of crypts contain only small clonal clusters. However, by 8 weeks, the majority of crypts have become resolved and information can be gathered on clustering. In the following, we have considered data from 8 and 16 weeks post-labelling. The raw data is shown in Figs. 3C and F. With four data points to constrain two parameters, p_0 and f, by making a fit of the ratio $P_{\mathrm{XX}}(t)/P_{\mathrm{X}}(t)$ and $P_{\mathrm{XY}}(t)/P_{\mathrm{X}}(t)$ to the experimental data, with $z = 6$, we estimate the crypt induction frequency to be around $p_0^{\mathrm{WT}} \simeq 0.006 \pm 0.002$ per crypt and the fission rate to be low at around $f^{\mathrm{WT}} = 0.01 \pm 0.002$ per eight weeks (Fig. 3G). At this rate, we can expect the vast majority of crypts to undergo zero rounds of fission over the life time of the animal. Moreover, with these parameters, the frequency of three-crypt clusters XXX is small with measured values consistent with the model, while larger clusters are both predicted and found to be absent even at 16 weeks post-induction.

For K-ras, the accelerated drift towards monoclonality allows additional measurements to made at 2 weeks post-induction. At this very early timepoint, the majority of clonal marked crypts have already expanded over more than 50% of the base region allowing them to be safely classified as monoclonal. In this case, from a fit to the data, we obtain $f^{\mathrm{Kras}} =$

0.27 ± 0.04 per 8 weeks, some 30 times larger than WT, while the induction frequency is comparable at $p_0^{\mathrm{Kras}} \simeq 0.008 \pm 0.002$ per crypt (Fig. 3H). (Note that, following induction, at the 16 weeks timepoint, only two colors were expressed. Fortunately, from the analysis above, one may see that, while $P_{\mathrm{XX}}(t)$, $P_{\mathrm{XY}}(t)$, and $P_{\mathrm{XXX}}(t)$ depend explicitly on the number of colors, n_{color}, the ratios, $P_{\mathrm{XX}}(t)/P_{\mathrm{X}}(t)$, and $P_{\mathrm{XXX}}(t)/P_{\mathrm{X}}(t)$ are independent allowing these values to be safely compared with theory without any adjustment. Since the ratio $P_{\mathrm{XY}}(t)/P_{\mathrm{X}}(t)$ scales as $(n_{\mathrm{color}} - 1)$ we expect the measured value to be a factor of 2 smaller than theory at 16 weeks, a prediction supported by the experimental data.) Significantly, the fit of the data at 8 and 16 weeks provides a good prediction of the observed frequencies at 2 weeks post-induction, lending further support to the validity of the model.

With these fits, we can further assess the ability of the model to predict the frequency of higher order clusters. The results are shown in the table below:

	8 weeks			16 weeks		
	expt.	model	error	expt.	model	error
XX	0.30	0.27	0.1	0.44	0.45	0.04
XXX	0.062	0.079	0.008	0.18	0.20	0.02
XXXX	0.029	0.013	0.003	0.031	0.072	0.014
XXXXX	0.002	0.003	0.002	0.006	0.030	0.009
XXXXXX	0	0.001	0.001	0.006	0.013	0.006
XXXXXXX	0	0	0	0.009	0.005	0.004

Although these findings show broad quantitative agreement of the model with the experimental data, at 16 weeks post-induction, the former has a tendency to slightly over-estimate the latter for the largest cluster sizes. Although this apparent departure may be a consequency of small number statistics, the discrepancy may also follow from the Markovian character of the approximation used for the modelling scheme. More precisely, in building the model of crypt fission, we have assumed that the timing between consecutive fission events is random and statistically uncorrelated. However, following fission, there may be an enforced delay between the next fission event while the Paneth cell numbers build and the stem cell compartment is regenerated in full. The impact of this "refractory" period will be particularly significant for the largest clusters, which rely on multiple rounds of fission. As a result, the theoretical model may provide a small overestimate of the tails of the distribution.

2.3 Crypt death

In the analysis of the data, we did not take account of the possibility that crypts may become lost altogether over time due to the loss of all stem cells from the crypt base. The association of stem cell competence with proximity to the Paneth cell niche suggests that crypt extinction due to the chance loss of all stem cells is unlikely. This view is corroborated by histological studies which show a lack of variability in the circumferential size of crypts near the base [1]. Nevertheless, even a low rate of crypt loss may be competitive with the low crypt fission rate in the wild-type tissue. Therefore, in this final section, we will return to consider the possible impact of crypt death, and whether the data can be used to estimate its potential frequency.

To implement this program, we must go back and reconsider the changes that can occur in crypt number over time when we account for both crypt fission and loss. In this case, the crypts now undergo a birth-death type process,

$$\mathrm{C} \mapsto \begin{cases} \mathrm{C}+\mathrm{C} & \mathrm{Pr.}\, f \\ \emptyset & \mathrm{Pr.}\, d \end{cases},$$

where, as before, f denotes the fission rate and d the loss rate. If, for simplicity, we assume that both of these events, crypt fission and death, follow a Poisson random process in which the time between consecutive events is again statistically uncorrelated, the chance that a labeled crypt has expanded to generate a cluster of n labeled crypts is given by (N. T. J. Bailey, The Elements of Stochastic Processes, J. Wiley & Sons, New York, 1964)

$$F_n(t) = \begin{cases} \frac{d}{f}\beta(t) & n = 0 \\ (1-\beta(t))\left(1-\frac{d}{f}\beta(t)\right)[\beta(t)]^{n-1} & n \geq 1 \end{cases}$$

where

$$\beta(t) = \frac{1-e^{-(f-d)t}}{1-(d/f)e^{-(f-d)t}}\,.$$

The survival probability of a single marked crypt is therefore given by

$$F^{\mathrm{surv.}}(t) = 1 - F_0(t) = 1 - (d/f)\beta(t)\,.$$

Therefore, if we focus on the distribution of "surviving" crypts, i.e. clusters that contain at

least one labeled crypt, then the probability is given by

$$g_n(t) = \frac{F_n(t)}{F^{\text{surv.}}(t)} = (1-\beta(t))[\beta(t)]^{n-1} \qquad n \geq 1\,.$$

Note that, in the absence of crypt death ($d = 0$), the distribution recovers the simpler form defined above. Moreover, for $(f-d)t \gg 1$ (which may, as before, translate to an unfeasibly long time), the distribution approaches an exponential scaling form,

$$g_n(t) = \frac{1}{\langle n(t)\rangle} e^{-n/\langle n(t)\rangle}\,,$$

where $\langle n(t)\rangle = \frac{1}{(1-d/f)} e^{(f-d)t}$ denotes the average cluster size.

With these results, we can now once again consider the leading order contributions to the cluster probabilities. However, in this case, the enterprise becomes even more daunting since any given crypt configuration can be reached by a multitude of different permutations involving crypt expansion by fission and loss by death. For example, consider the chance that, after a time t post-labelling, a cluster will involve a single isolated labelled crypt. Clearly, such an event could arise from multiple sources. In the first place, over the timecourse, a single marked crypt may have undergone a history in which its number ended up unchanged (either by not expanding at all, or by transiting through an equal number of fission and death events). This would contribute factor of $n_{\text{color}} p_0 \times \frac{F_1(t)}{F^{\text{surv.}}(t)}$ to the probability. Here we must use a normalization, $F^{\text{surv.}}(t)$, that includes all possible channels of loss starting from an arbitrary initial configuration of marked crypts. Taking into account all initial conditions, one crypt, two, etc., we have that,

$$F^{\text{surv.}} = 1 - n_{\text{color}} p_0 F_0(t) - n_{\text{color}} z p_0^2 F_0^2(t) - n_{\text{color}}(n_{\text{color}}-1) z p_0^2 F_0^2(t) - \cdots$$

Alternatively, we can arrive at a single marked crypt from the net extinction of one crypt from an induced pair leading to a contribution of $n_{\text{color}} z p_0^2 \times 2\frac{F_0(t)F_1(t)}{F^{\text{surv.}}(t)}$, where the factor of two accounts for the multiplicity. Similarly, we gain a further contribution from a process in which two out of three initially marked crypts become extinct leading to a contribution $n_{\text{color}} \frac{1}{2} z(z-1) p_0^3 \times 3\frac{F_0^2(t)F_1(t)}{F^{\text{surv.}}(t)}$, and so on. Equally, a single marked crypt could follow from the extinction of one of the marked crypts in a neighboring pair of different color, leading to a contribution $n_{\text{color}}(n_{\text{color}}-1) z p_0^2 \times \frac{F_0(t)F_1(t)}{F^{\text{surv.}}(t)}$, and so on and so forth.

However, while the induction probability, p_0, and effective rate constant $(f-d)$, remain small, the leading contribution will arise from the first of these processes (chance induction

following by "quiescence"), viz.

$$P_{\rm X}(t) \simeq n_{\rm color} p_0 \times F_1(t)\,,$$

with $F^{\rm surv.} \simeq 1$.

Similarly, if we consider the probability of two marked neighboring crypts to have different color, although there are many potential contributions, the dominant one will arise from the contribution in which the net number has not changed from the initial condition, i.e.

$$P_{\rm XY}(t) \simeq n_{\rm color}(n_{\rm color}-1) z p_0^2 \times F_1^2(t) = (n_{\rm color}-1) z p_0 F_1(t) P_{\rm X}(t)\,.$$

Turning now to the cluster probability of a common color, let us consider chance of finding two neighboring crypts. Once again, such an event could arise from multiple sources. First, it can arise from the net fission of a single marked crypt, with a probability, $n_{\rm color} p_0 \times \frac{F_2(t)}{F^{\rm surv.}(t)}$. Second, this configuration can follow from the induction of two neighbouring crypts followed by a zero net increase in the size of the cluster, leading to a contribution $n_{\rm color} z p_0^2 \times \frac{(F_1^2(t)+2F_0(t)F_2(t))}{F^{\rm surv.}(t)}$. Finally, we can obtain two marked crypts through a sequence of extinction events from clusters of larger size and/or broader composition. However, once again, while the induction probability, p_0, and rate $(f-d)$, remain small, the leading contribution will arise from the first two of these processes, viz.

$$P_{\rm XX}(t) \simeq n_{\rm color} p_0 \times \frac{F_2(t)}{F^{\rm surv.}(t)} + n_{\rm color} z p_0^2 \times \frac{F_1^2(t)}{F^{\rm surv.}(t)} = \left(\frac{F_2(t)}{F_1(t)} + z p_0 F_1(t)\right) P_{\rm X}(t)\,.$$

Similarly, taking only the leading contributions, we have the higher order cluster probability,

$$\begin{aligned} P_{\rm XXX} &\simeq n_{\rm color}\frac{1}{2} z(z-1) p_0^3 \times \frac{F_1^3(t)}{F^{\rm surv.}(t)} + 2 n_{\rm color} z p_0^2 \times \frac{F_1(t)F_2(t)}{F^{\rm surv.}(t)} + n_{\rm color} p_0 \times \frac{F_3(t)}{F^{\rm surv.}(t)} \\ &= \left(\frac{F_3(t)}{F_1(t)} + 2 z p_0 F_2(t) + \frac{1}{2} z(z-1) p_0^2 F_1^2(t)\right) P_{\rm X}(t)\,. \end{aligned}$$

On this background, we can consider whether crypt death plays an important role in the unfolding of the crypt clonal dynamics. With only two timepoints for the wild-type system, it is impractical to attempt to estimate the two rate constants and the induction frequency from the experimental data. However, we can estimate the potential impact of crypt death by turning to an extreme limit where $d \gg f$. In this case, the dynamics converges rapidly onto

a pure death process in which

$$F_n(t) = \begin{cases} 1 - e^{-dt} & n = 0 \\ e^{-dt} & n = 1 \\ 0 & n > 1 \end{cases} .$$

In this case, it is evident that both $P_{\mathrm{XY}}(t)/P_{\mathrm{X}}(t)$ and $P_{\mathrm{XX}}(t)/P_{\mathrm{X}}(t)$ scale in proportion to $F_1(t) = e^{-dt}$ and would be predicted to undergo a progressive decay. Such behavior is easy to understand as any initial configuration must eventually pass through a state with a single marked crypt before undergoing extinction altogether. However, such decay is at odds with the experimental data which show that, even for the WT system where the fission rate is low, the frequency of XX clusters steadily rises over time, while the frequency of XY clusters remains approximately constant. Although we are not able to rule out a death rate altogether, we can conclude it is as best comparable and likely smaller than the fission rate of crypts even in the wild-type system. Extrapolated to the K-ras mutant system, where the fission rate some 30 times larger than wild-type, it is clear that the impact of crypt death may be neglected in the analysis of the data.

2.4 Comparison with previous studies

Finally, previous studies have attempted to estimate the rate of crypt fission in normal tissue. In particular, 25 years ago, Totafurno *et al.* introduced a model involving the continuous growth and bifurcation of crypts through fission [4]. Based on the analysis of crypt burfurcations, they introduced a model based on the notion of a continuous and regular crypt cycle and concludes a crypt fission rate of around once per 15 weeks, a factor of 50 higher than that predicted by this work, and greatly in excess of that found even for K-ras mutant crypts. It is evident that these findings are inconsistent both with the analysis presented in this work and with the clonal fate data reported here. However, since the model discussed by Totafurno *et al.* differs fundamentally from that considered in this work, it is difficult to comment on the potential source of discrepency.

In a second more recent study, Li *et al.* made use of a clonal fate study using a mutation-induced marker system to infer a crypt "cycle time" [5]. From a study of the average clone size of crypts, using a model similar to the one implemented here, they deduced a crypt fission rate in control animals of around $f = 0.03$ per eight weeks, a factor of three larger than that estimated here. Given the potential for variation by mouse strain, position within the

small intestine, and toxicity of the mutagen, the coincidence of these small rates is surprisingly good.

References

1. Lopez-Garcia C, Klein AM, Simons BD, and Winton DJ (2010) Intestinal stem cell replacement follows a pattern of neutral drift. *Science* **330**: 822-825

2. Snippert HJ, van der Flier LG, Sato T, van Es JH, van den Born M, Kroon-Veenboer C, Barker N, Klein AM, van Rheenen J, Simons BD, and Clevers H. Intestinal crypt homeostasis results from neutral competition between symmetrically dividing Lgr5 stem cells. *Cell* **143**: 134-144

3. Kozar S, Morrissey E, Nicholson AM, van der Heijden M, Zecchini HI, Kemp R, Tavar S, Vermeulen L, and Winton DJ (2013) Continuous Clonal Labeling Reveals Small Numbers of Functional Stem Cells in Intestinal Crypts and Adenomas. *Cell Stem Cell*, in press.

4. Totafurno J, Bjerknes M, and Cheng H (1987) The Crypt Cycle: Crypt and Villus Production in the Adult Intestinal Epithelium. *Biophys. J.* **52**: 297-294

5. Li YQ, Roberts SA, Paulus U, Loeffler M, and Potten CS (1994) The crypt cycle in mouse small intestinal epithelium. *J Cell Sci.* **107**: 3271-3279
