# Supplementary material for: Biased competition between Lgr5 intestinal stem cells driven by oncogenic mutation induces clonal expansion
Source: EMBO Rep. 2013 Dec 16;15(1):62–9. doi: 10.1002/embr.201337799 (PMC3983678; doi:10.1002/embr.201337799)
Supplement: Supplementary file 4 [file embr0015-0062-sd4.pdf]

EMBO
reports

Manuscript EMBOR-2013-37799

Biased competition between Lgr5 intestinal stem cells driven by oncogenic mutation induces clonal expansion

Hugo J. Snippert, Arnout G. Schepers, Johan H. van Es, Ben D. Simons and Hans C. Clevers

Corresponding authors: Hugo J. Snippert and Hans C. Clevers, University Medical Center Utrecht

Review timeline:

Submission date:	23 July 2013
Correspondence:	13 August 2013
Correspondence:	14 August 2013
Correspondence:	15 August 2013
Editorial Decision:	15 August 2013
Revision received:	09 October 2013
Editorial Decision:	05 November 2013
Revision received:	06 November 2013
Accepted:	06 November 2013

Transaction Report:

(Note: With the exception of the correction of typographical or spelling errors that could be a source of ambiguity, letters and reports are not edited. The original formatting of letters and referee reports may not be reflected in this compilation.)

Editor: Nonia Pariente

Correspondence - Editor 13 August 2013

I have now received the comments from the three referees that were asked to assess your study, which you will find at the end of this email.

As you will see, they raise a number of issues and -as I mentioned in our earlier correspondence- given your competitive situation, it would only make sense to invite revision if it could be completed within a reasonably short time frame. Thus, before making a final decision, it would be helpful to know how you would be able to address the referee concerns and the amount of time you would need to do so.

I appreciate that the second major issue raised by referee 3, regarding the mechanism behind mutant KRas-driven stem cell expansion, is out of the scope of a short report. Nevertheless, other issues raised by this referee and specially referee 1 concern central claims of the study and cannot be dismissed.

© European Molecular Biology Organization

However, as I understood, you have had these data for some time and perhaps the mice have aged sufficiently to address some of referee 1's concerns by analyzing later time points?

In order not to delay the decision-making process, please respond within 48 hours. I look forward to hearing from you.

REFEREE REPORTS:

Referee #1:

I like the ideas behind this article and think that the experiments are elegant.

However, I am concerned about z number of things, which I detail below.

1) I think that by and large the section on clonal expansion of k- ras mutated stem cells by biased drift is well thought out. However leaving aside the fact that, like Henry Harris, I dislike claims of being the first to do something (which should be for others to decide), it is not true that this is the first time that crypt fission has been shown to be responsible for clonal expansion in the intestine. While you have quoted some of the literature on this, notable by its absence are two articles by Matthew Bjerknes - and in particular the J Theor Biol paper "Expansion of mutant cell populations in the human colon" 1996;178: 381. In this he shows that cluster size analysis shows that aberrant crypt populations which have k-ras mutations (or APC) expand faster (interesting enough 40 times faster) than normal crypts - by fission. The other paper is Proc R Soc Biol 1995: 260; 1-6 on crypt cycle asymptotic dynamics.

In addition, as far as I can see, you don't seem to have validated that the efficiency with which the brainbow reporter and the KRAS transgene are co-recombined. There may be many instances of labelled cells that are KRAS wild-type, a consequence of which, I believe, is that the effect size of the KRAS mutants is underestimated.

The live imaging data of Ritsma sounds compelling and is central to the argument presented, and so could do with some more explanation.

A supplementary figure showing the suggested insensitivity of the results to the stem cell number would be welcome.

The estimated loss-replacement rate of 0.25/day is significantly less than the estimates previously proposed by the authors (approximately 1/day, or 0.75/day depending on the model the authors used). Presumably, some of this difference is accounted for by the definition of the stem cell pool used in this work (N=8). However, it is not immediately obvious to me how these differences scale with the stem cell number. An acknowledgement and discussion of this issue would be good to see.

2) A further point is that you have not shown field cancerization. A group of 8 crypts is just clonal expansion, the sort of size you would get in an aberrant crypt focus, and I do not think this constitutes a 'field'. Of course it is probable that, as has been discussed at lengths many times (cf. Garcia S et al "Field cancerization, clonality and epithelial stem cells: the spread of mutant clones in epithelial sheets". J Pathol 1999: 186: 61-81), crypt fission is the underlying cause of field cancerization, it is just that you have only shown relatively minor clonal expansion. You would have to look at longer time points for this - I assume that this is possible?

The data for the crypt fission rate is rather sparse and belies confidence in the conclusions reached in this section. The length of the longest chase period for assessing the rate of crypt fission is rather short (only 16 weeks), and data has only been assessed at two time-points. Assessing the general trend in the data, and that the model fits it, is difficult to judge from these data. Indeed, the model is often outside of the SEM for the data. Showing a much longer chase period - or preferably multiple longer chase periods - would greatly improve confidence in the fit of the model as well as show field cancerization, as indicated above.

3) The analysis does not include the possibility of crypt death -including this could really change the estimate of the crypt growth rate- and the data might be sufficient to accept or reject crypt death as a common phenomenon. The issue of whether crypt death is a common, and indeed normal, occurrence is contentious and of interest to the field. As such a birth-death process may be a more suitable model of the data. There may also be sufficient information in the clone size data to reject the birth-death process in favour of a birth-only process - if so this would be a significant result. Is this something that the mathematical analysis could assess? Finally, a consideration of crypt death may lead to more tangible hypothesises regarding the mechanism driving crypt fission.

The estimates of the crypt fission rate are significantly lower than previous direct estimates:
Li, et al. (1994). The crypt cycle in mouse small intestinal epithelium. Journal of cell science, 107 (Pt 12), 3271-3279.
Totafurno, J., Bjerknes, M., & Cheng, H. (1987). The crypt cycle. Crypt and villus production in the adult intestinal epithelium. Biophysical journal, 52(2), 279-294.
This should be discussed. The reviewer imagines that the fitting of a birth-death process could lead to larger estimates of the crypt fission rate.

4) The authors' study is predicated on the assumption that crypts follow a canonical crypt cycle that has a regular (average) duration. Do the authors consider that they have evidence for this hypothesis? Or is their data better represented by random induction of fission due to some local stimulus (eg no characteristic timescale for the life-cycle)?

Minor:

Abstract: The phrase "mutant tissue expands" is a bit funny. Just describe it as clonal expansion?

Concept of fitness in the introduction. You could be specific as to what phenotypes increase fitness. Eg., a proliferation or survival advantage, or something to do with spindle orientation.

Results, section on Paneth cells in crypt fission. A p-value should be provided to justify the statement of equal numbers of Paneth cells in the two crypt branches. A binomial test is likely appropriate.

Referee #2:

Experimentally this is an elegant study that makes use of the possibility of labelling and tracing individual stem cells to study the effects of an oncogenic ras mutation in the stem cell compartment and at following stages. Results suggest that ras increases the rate of drift to clonality within the stem cell compartment and also increase the rate of crypt fission which would allow spreading of the oncogenic mutational in line with the concept of field cancerization. In fact this is what one would expect if ras endows cells with a growth advantage over wild type cells but it has not been studied before in a similar manner as here. Leaving aside the mathematics that needs to be reviewed by an expert, several points however are not clear to this reviewer because there are some gaps in the description of the study design and interpretation of results. Comments below should serve as suggestions to clarify certain points of this otherwise elegant study.

Unclear points

1 The scheme in Fig. 1 indicates that the loss of stem cells through differentiation and replacement by neighbouring stem cell leads to the domination of one stem cell over the others ultimately resulting in a clone of stem cells in a given crypt. If authors restrict their confocal investigations to the level of the stem cell compartment as suggested by the scheme why are Lgr5 high cells i.e. stem cells declining after tracing? Shouldn't all cells at this level be LGR5 positive? What is the nature of the other cells in the "clone"? Are they differentiated but still remain at this position of the crypt?

2 Related to point 1: The authors state that in the K-ras mice more Lgr5 positive cells "survive" than in the WT control. What is meant by surviving? Does it mean that ras prevents stem cells from developing progeny towards the transit amplifying compartment? Wouldn't that rather limit spreading of the mutation?

3 It is suggested that this study reveals in vivo insights to the field cancerization. This appears to be quite evident by the finding that in K-ras mice there is an increased rate of crypt fission which will allow the spreading of the mutation. However, at the levels of stem cells this is less clear because in the experimental setting all stem cells should have the mutant K-ras expressed and no competition between normal and mutated cell can take place. Thus; what is observed is a faster "drift to clonality" i.e. faster loss and replacement among K-ras mutated cells. One cannot predict whether mutated stem cells would out-compete wild-type stem cells in a mosaic situation. If for instance K-ras acts in a non cell autonomous fashion, e.g. by secretion of growth factor it might also promote drift to clonality of wild type cells.

4 In that context what is meant by the term "survival probability" of a K-ras mutant cell mentioned on p. 5 first line?

Referee #3:

The concept of field cancerization is well known in epithelial cancers particularly the skin. In this report Snippert and colleagues suggest KRAS mutations lead to field cancerization in the intestinal epithelium.
Overall the experiments performed are well, the data analysed correctly and it is clear KRAS mutations have an advantage over wild type stem cells and then Kras mutations are further expanded by crypt fission.
The questions though that remain however is
1) is this relevant for human CRC, most data have shown the p53 mutations spread in colitis(the elegant work of the Wright group) there is very little evidence of spreading of KRAS mutations.
2) There is no mechanism here of how KRAS mutation does this. At least some mechanistic work could be done ie if Lgr5CREER KRASG12D mice are treated with MEK inhibitors does this suppress the stem cell expansion.
It will be important for the authors to attempt to address these questions as the paper currently in my opinion is a little too descriptive.

Other points
1) The mutation of human CRC show APC/b-catenin mutations at 80% and KRAS mutations at 40%. If KRAS mutations do spread first one would expect that sequencing of different places of the the same tumours would show concurrent APC and KRAS mutations. Does published work show this (eg Work of the Wright group)?.
2) Given the authors have done Lgr5 APCfl/fl do they see the same increase in clone sizes and crypt fission (over the 14 day timepoint) prior to adenoma formation.
3) Is the same KRASG12D effect seen in the colon? A much greater effect of this allele is seem when it is targeted to the colon (Haigis paper Nature Genetics)
4) Better citing of the working where KRASG12D mutation has been targeted to the mouse intestine is required. For example data has already been published suggesting KRAS does not alter the stem cell pool
Feng Y, Bommer GT, Zhao J, Green M, Sands E, Zhai Y, Brown K, Burberry A, Cho KR, Fearon ER. Mutant KRAS promotes hyperplasia and alters differentiation in the colon epithelium but does not expand the presumptive stem cell pool.
Gastroenterology. 2011 Sep;141(3):1003-1013
Interesting this paper shows that differentiation is altered as did the Haigis paper. This should be discussed at the very least. It is interesting to note that this paper also shows that KRAS mutation causes a loss of paneth cells over the long term. This again should be discussed. Could KRASG12D stem cells be more likely to stay as stem cells and not produce a secretory/paneth cells offspring?

Discussion of this data is also important given the discussion of the paneth cells in the paper.
5) Quantification for figure 1 should be provided on the figure with statistics.

Correspondence – Authors response 14 August 2013

Thank you for your time, considerations and efforts to help our manuscript.

We are encouraged by the favorable comments of the referees and note that two of the reviewers refer to the elegance of the study. Indeed, we are confident that the majority of the important issues raised by the reviewers, such as relevance for human CRC, can be addressed in full through a careful re-writing of the manuscript, and further clarification of our findings.

However, the referees raise several more substantial points on which we wish to draw your attention:
First, we have data on the co-recombination of the confetti and the k-ras alleles (point 1, ref 1), to which we can return and re-analyze to address the degree of underestimations of the actual effects.

Second, referee 1 raises the question of whether we can provide additional data to support our analysis of crypt fission. Clearly, we could, in principle, undertake a further long-term study of the clonal dynamics extending to timescales of 24 weeks and more. However, as you are aware, since there is pressure from a study by a competitive group, we are somewhat reluctant to embark such a lengthy program as we do not have aging mice. Moreover, the concern is actually about the validity of the model. We have now analyzed data from a third early time point (2wks tracing), which fits well with the predictions of the fission dynamics. This strengthens the model and provides further evidence for its validity.
Please note that the lines in the graphs are no simple fits. Instead, these follow from a model that is based on the cluster distribution at one time point. Subsequently, the model gives a very good prediction for cluster types at other time points. We will rewrite the manuscript and strengthen the point that two of the three timepoints are correctly predicted by analysis of the third.

Moreover, until now we have only analyzed the distribution of the smaller sized clusters (up to 3 crypts). We will now further challenge the model by examine the higher sized clusters as well. It is an aspect of the data sets which we reported on, but did not yet analyze quantitatively in the framework of the model. In our knowledge, this will strengthen the confidence in our model even further.

Last, as suggested in your e-mail, we believe that the elucidation of further mechanistic insights might reach beyond the scope of a short report. Nevertheless, I managed to obtain few mice that can be utilized to address faster cell cycle behavior of K-ras stem cells. If desired, such experiments might provide more insights into the underlying mechanism of competitive advantage of K-ras stem cells, although the downside is that these require more time to perform.

On the basis of the proposed analysis and revisions of the manuscript, we believe that we can re-submit the manuscript within two to three weeks.

Correspondence - Editor 14 August 2013

Thank you for your detailed email. Before we make a final decision, I wanted to receive your feedback on two further points. I feel there has been a misunderstanding of the issue raised by referee 1 in his/her point 2. What is of most concern to us is the argument that you have not shown field cancerization -which is a central claim of the study- but "only a limited clonal expansion". It is a pity that you don't have aging mice to address this in the manner the referee suggests; do you think there is anything you could do to address this specific concern that would not entail a 24 week project? I note you propose to strengthen the model, which would address the last part of ref 1's point 2, but not quite respond to the fact that you don't see "fields".

I am also unsure from your response if you would be in a position to incorporate and modify your

model to assess whether crypt death is a factor to consider, and whether this modifies the estimates of the crypt fission rate.

I think it would be reasonable to consider a revision period of 7-8 weeks, which would give you some more time to address the referee concerns.

Please let me know what you think as soon as possible, as we aim to make a decision on the study before the end of the week.

Correspondence – Authors response 15 August 2013

My apologies for this somewhat late reply. Last night I contacted Ben Simons and Hans Clevers to discuss the two topics that you brought up. Please find below our discussions on both of your questions. I hope these answer your questions sufficiently. For more questions, please do not hesitate to contact me.

With respect to your first question, we truly believe that we have shown the principles of field cancerization. The commonly use definition of it is 'expanding clones of genetically altered, but morphologically normal cells, that increase the risk for cancer development'. We clearly show expanding clones of K-ras, a mutation known to progress adenomas towards aggressive adenocarcinomas and increase the target size of cells (cells-of-origin) where cancer development can start. Moreover, the clones are histologically undetectable in the small intestine.
We think it becomes a discussion about semantics of what to call a 'field' and what not. Importantly however, as referee 1 already commented, the strength of our manuscript are insights on how field cancerization starts (via biased drift, followed by crypt fission) and not how large field of mutant cells behave in and possibly disturb epithelial homeostasis.

Moreover, there are two more points to emphasize on this subject. First, following K-ras activation in one stem cell, within 3 months the number of K-ras mutant stem cells in 8 monoclonal crypts has increased by a factor of 120, while the total number of mutant crypt progenitor cells has expanded by a factor of 2000!
Second, according to the dynamics of the process, the average cluster size increases exponentially fast. Of course, as there is a distribution of clone sizes, it makes no sense to speak of a "largest" clone (nor to predict it). But, rather, from the rates inferred from this study, we can predict that at 1 year post-induction, some 10% of surviving K-ras clones would have a size larger than 16, and 5% have a size larger than 21. If we translate these rates to human, such effects could lead to the development of macroscopic lesions of K-ras mutant cells over years, consistent with the evidence for field cancerization found from studies of somatic mitochondrial DNA mutations in human patients with Crohn's disease and intestinal neoplasia (Galaduik et al., Gastroenterology, 2012). We, therefore, maintain that the current study provides significant new insight on processes that contribute to the development of field cancerization.

With respect to crypt deaths, indeed, in our analysis of crypt fission, we have not yet taken into consideration the potential for crypt death either in the WT or K-ras mouse model. In a revised manuscript, we can address

this issue in a rigorous manner. However, we can already comment on the scale and significance of crypt death in relation to the conclusions of the study.

First, in respect of WT clones, we have found a good agreement of the model with the range of experimental data without taking into account the potential for crypt death. Moreover, our WT fission rate is broadly in line with that obtained in the Li et al. study to which the reviewer refers (despite a comment to the contrary made by the reviewer). Crucially, if crypt death contributed significantly, i.e. with a death rate that was comparable to the WT fission rate, we would not expect to obtain such a correspondence.

In respect of the K-ras data, would we assume that the crypt death rate was the same (apparently low value) as WT, it is clear that its contribution would be even more negligible when compared against the very high fission rate. However, it could be that the crypt death rate, along with the fission rate, is proportionately increased following K-ras activation. In this case we can make two rather technical remarks: First, as with WT, even without crypt death, a fit to the 8 week time point provides an excellent prediction of the 16 week time point suggesting that the crypt death, if present at all, contributes at a rather low rate. Second, at longer times, when the cluster size distribution becomes dominated by fission events as opposed to the chance induction of neighbouring crypts, the predicted size distribution of a birth-death process formally coincides with that of a pure birth process. In short, the distribution depends only on the net difference between the birth and death rates. In this case, the quantitative analysis would formally coincide (in the sense that the mathematical predictions would be the same), but the interpretation of the rates would be adjusted accordingly. Indeed, from a practical point of view, it is of course the net difference between crypt fission and loss that matters from the perspective of field cancerization.

In summary, the reviewer has raised an important question on crypt birth and death rates. For the reasons outlined above, we do not believe that the magnitude of the crypt death rate, even if it is non-zero, is large enough to impact on our findings. However, we agree that it is important to address this point carefully and explicitly in the manuscript.

1st Editorial Decision 15 August 2013

I have now had time to read your emails and discuss the decision within our editorial team, as well as to receive further input from the referees. From all of these discussions, it is clear that there is sufficient interest to invite a revision of your study.

That said, referees 1 and 3 maintain their concerns, and referee 3 agreed that whether the clonal expansions can become fields is an important issue. Whether or not K-Ras mutations lead to field cancerization is a critical point. Upon further discussion, referee 1 is concerned that you have shown thus far a limited clonal expansion and not a field change. S/he considers that what you have described is about the size of an aberrant crypt focus (ACF) and it may well be that k-ras mutant crypt patches can't get much bigger. It appears that in human colon, dysplastic ACFs show k-ras mutations but are usually of strictly limited size: in the absence of evidence to the contrary, which s/he believes necessary if you want to be convincing, s/he favours the view that k-ras mutations alone give rise only to limited clonal expansion, and that field cancerization would need further mutations.

However, as you mentioned in your first email that you would be able to address the concern

regarding the relevance of your results for human CRC, this may also somewhat alleviate the concern regarding cancerization. If you cannot convincingly show that what you see is the initial stage of field cancerization, the conclusions in this respect would have to be toned down and all these issues openly discussed.

Revision would also entail addressing the other points brought up by the referees, including a strengthening of the model by analyzing a third time point, and examining higher sized clusters, as you mentioned, as well as including crypt death. I do think that incorporating the analysis of the cell cycle behaviour of K-Ras mutant cells would be a first step towards a mechanism that would strengthen the study, given the circumstances. Please note that it is EMBO reports policy to undergo one round of revision only and thus, acceptance of your study will depend on the outcome of the next, final round of peer-review.

Revision will also entail re-writing of the manuscript, as well as inclusion and discussion of several references. Please do keep in mind that the text cannot be excessively lengthened, as it is already longer that we can normally accommodate. Shortening may be made easier by combining the Results and Discussion into a single section, which we require, and which will help eliminate the redundancy that is inevitable when discussing the same experiments twice. Please note that basic Materials and Methods, required for understanding the experiments performed, must remain in the main text and thus this section cannot be any shorter.

Your revised manuscript should be submitted within 8 weeks. It will otherwise be treated as a new submission and editorially evaluated with respect to novelty at the time of submission (if still sufficiently novel, it would be sent to the same referees).

When submitting your revised manuscript, please include:

a Microsoft Word file (or equivalent) of the manuscript text, editable TIFF or EPS-formatted figure files, a separate PDF file of any Supplementary information (in its final format) and a letter detailing your responses to the referee comments.

We would also welcome the submission of cover suggestions, or motifs to be used by our Graphics Illustrator in designing a cover.

I look forward to seeing a revised form of your manuscript when it is ready. In the meantime, do not hesitate to get in touch with me if I can be of any assistance.

1st Revision - authors' response 09 October 2013

Referee #1:

The reviewer raises a number of important concerns, which we have sought to address. In the following, we detail the remarks of the reviewers, and the steps that we have taken to address the points.

1) I think that by and large the section on clonal expansion of k- ras mutated stem cells by biased drift is well thought out. However leaving aside the fact that, like Henry Harris, I dislike claims of being the first to do something (which should be for others to decide), it is not true that this is the first time that crypt fission has been shown to be responsible for clonal expansion in the intestine.

Following the advice of the referee, we have acknowledged previous attempts to study the problem of crypt fission and have suitably adjusted the narrative accordingly. We are grateful to the referee for highlighting the literature below.

While you have quoted some of the literature on this, notable by its absence are two articles by Matthew Bjerknes - and in particular the J Theor Biol paper "Expansion of mutant cell populations in the human colon" 1996;178: 381. In this he shows that cluster size analysis shows that aberrant

crypt populations which have k-ras mutations (or APC) expand faster (interesting enough 40 times faster) than normal crypts - by fission. The other paper is Proc R Soc Biol 1995: 260; 1-6 on crypt cycle asymptotic dynamics.

We apologize for overlooking these two papers which are obviously germane to the conclusions drawn in this work. Both are now included in the manuscript:

'Although the rate of crypt fission in steady state epithelium is low, the frequency can increase as a regenerative response to epithelial injury or due to mutations [25-28].'

In addition, as far as I can see, you don't seem to have validated that the efficiency with which the brainbow reporter and the KRAS transgene are co-recombined. There may be many instances of labelled cells that are KRAS wild-type, a consequence of which, I believe, is that the effect size of the KRAS mutants is underestimated.

The referee is correct in noting that failure of co-recombination could lead to an underestimate of the effect of K-ras mutation on both the degree of bias and the rate of crypt fission. These false positives and false negatives have now been quantified, acknowledged in the main text and specified in the supplementary information. However, we note that the effect of false positives (oncogenic K-ras but not marked with the Confetti) is mitigated by the fact that such events in otherwise unlabeled clones would escape detection. Similarly, the majority of false negatives (Confetti marked but not K-ras activated) would fail to drift to monoclonality and would therefore not contribute to the longer-term fission data. Indeed, the consistency of the predictions of the modeling scheme with the experimental data suggests that such events do not significantly corrupt the data. That said, the reviewers is quite correct that such effects may lead to an underestimate of both the degree of bias and the fission rate in K-ras mutant clones. As such, we have included the following text:

'Indeed, given that Cre-recombination efficiency shows a degree of variability between genetic loci, it is likely that this analysis provides a small underestimate of the true scale of bias (Sup. info).'
Sup. Info: 'Cre-recombination efficiency shows a degree of variability per genetic locus. Therefore, clones exist that express oncogenic K-ras but that are not marked with Confetti (false negatives, i.e. 169 clones/ 2000 crypts) and vice versa (false positives, i.e. 8 clones/ 2000 crypts, versus 383 true positives/ 2000 crypts). Both scenarios in fact skew the data towards an underestimation of true scale of the bias.'

The live imaging data of Ritsma sounds compelling and is central to the argument presented, and so could do with some more explanation.

Following the advice of the referee, we have extended and clarified the description of the Ritsma paper. We hope the referee can forgive us for leaning towards brevity to conform to the space limits of the journal. However, we note that a recent paper from the Winton lab (referenced in the text) lends further support to the conclusion of the unpublished Ritsma paper. Accordingly, we have made the following revision:

'Recent studies based on multi-day imaging of lineage traced clones that are initiated in Lgr5 intestinal stem cells, suggest that stem cells positioned near the boundary of the niche are temporally biased towards displacement from the niche and loss of stemness, suggesting the effective stem cell number is less than the number of $Lgr5^{hi}$ cells (Ritsma et al., unpublished). These findings are corroborated by a recent lineage tracing study by Kozar et al. that defines a small "functional" stem cell population [22]. Here, based on the quantitative analysis of Ritsma et al., we take a figure of N=8, noting that, while we are concerned with the relative difference between WT and K-ras mutant cells, our conclusions are largely insensitive to the precise choice.'

A supplementary figure showing the suggested insensitivity of the results to the stem cell number would be welcome.

The origin of the insensitivity to stem cell number follows from the form of the clone size distribution following neutral or biased drift dynamics. In both cases, the system is predicted to enter a scaling regime where the size distributions depend only the ratio of loss/replacement rate to the square of the effective stem cell number (see Lopez-Garcia et al, and the supplementary materials).

© European Molecular Biology Organization

Adjustment of one these parameters can be compensated by adjustment of the other leading to the same parametric dependence. This insensitivity implies that, while the ratio may be reliably inferred from the data, the error bar on the individual parameters is large. Fortunately, our interest here lies in the comparison of the WT and K-ras data. In this case, we may focus on the relative increase of the loss/replacement rate, which can be inferred reliably regardless of the particular value of the effective stem cell number.

The estimated loss-replacement rate of 0.25/day is significantly less than the estimates previously proposed by the authors (approximately 1/day, or 0.75/day depending on the model the authors used). Presumably, some of this difference is accounted for by the definition of the stem cell pool used in this work (N=8). However, it is not immediately obvious to me how these differences scale with the stem cell number. An acknowledgement and discussion of this issue would be good to see.

This discrepancy is a reflection of precisely the issue discussed above. Indeed, the consistency of the analysis and the explanation of the difference is central to the discussion of the Ritsma et al. paper. We have acknowledged this point more precisely in the supplementary materials section.

2) A further point is that you have not shown field cancerization. A group of 8 crypts is just clonal expansion, the sort of size you would get in an aberrant crypt focus, and I do not think this constitutes a 'field'. Of course it is probable that, as has been discussed at lengths many times (cf. Garcia S et al "Field cancerization, clonality and epithelial stem cells: the spread of mutant clones in epithelial sheets". J Pathol 1999: 186: 61-81), crypt fission is the underlying cause of field cancerization, it is just that you have only shown relatively minor clonal expansion. You would have to look at longer time points for this - I assume that this is possible?

We do acknowledge the criticism of the referee that what we have showed thus far might be considered clonal expansion only. We have therefore lowered the tone in our manuscript to report the phenomenon as clonal expansion. More importantly we substituted the phrase ‘field cancerization’ in the title with ‘clonal expansion’ and further down the manuscript we removed direct references towards the phenomenon of field cancerization.

Nevertheless, we consider the phenomena that we describe in our manuscript (biased drift followed by crypt fission) as providing a platform on which field cancerization can operate, providing a mechanism that can explain how mutations that do not visibly alter tissues can still drive clonal expansion.

Finally, the referee quite reasonably questions what happens at times longer than the 16 week chase reported here. Unfortunately the littermates that we set up in the past for longer time points all died. This was presumably due to activation of oncogenic K-ras in multiple Lgr5 stem cell compartments in the mouse, but this remains speculation.

The data for the crypt fission rate is rather sparse and belies confidence in the conclusions reached in this section. The length of the longest chase period for assessing the rate of crypt fission is rather short (only 16 weeks), and data has only been assessed at two time-points. Assessing the general trend in the data, and that the model fits it, is difficult to judge from these data. Indeed, the model is often outside of the SEM for the data. Showing a much longer chase period - or preferably multiple longer chase periods - would greatly improve confidence in the fit of the model as well as show field cancerization, as indicated above.

We acknowledge that further evidence in support of the model would be desirable. However, as detailed above, we cannot provide data for a longer chase period, largely for technical reasons. However, to strengthen our analysis, we have now included data from a 2 week time point, which can be achieved due to the rapid conversion of crypts in which oncogenic K-ras has been activated. Further, we have now analyzed the largest clones at the longest time point. In both cases, we find that the model, whose parameters are fully determined by just two data points at one time point, can accurately predict the behaviour from 2 weeks to 16 weeks. We believe that this provides compelling evidence for the validity of the modeling scheme.

3) The analysis does not include the possibility of crypt death -including this could really change the estimate of the crypt growth rate- and the data might be sufficient to accept or reject crypt death as

a common phenomenon. The issue of whether crypt death is a common, and indeed normal, occurrence is contentious and of interest to the field. As such a birth-death process may be a more suitable model of the data. There may also be sufficient information in the clone size data to reject the birth-death process in favour of a birth-only process - if so this would be a significant result. Is this something that the mathematical analysis could assess? Finally, a consideration of crypt death may lead to more tangible hypothesises regarding the mechanism driving crypt fission.

We acknowledge that, in the original version of the manuscript, we did not consider the important question of crypt death. Since stem cell number appears to be fixed by niche size and, from histological sections, appears to fluctuate little between crypts, we felt that crypt death was an unlikely event. However, it is very reasonable to question whether the clonal data can provide independent evidence in support of this conjecture. To address this point, we have now included a section on crypt dynamics including the potential for crypt death. From the analysis of this "birth-death" process, it is evident that, when the rate of death becomes comparable to fission, the ratio of XX type crypts to X crypts would diminish over time. By contrast, we find that the frequency of such events increases even for the WT system, consistent with the death rate being significantly smaller than the fission rate. Since the fission rate for K-ras active crypts is a factor of 30 larger than WT, we conclude that its effect on the K-ras data, if it is present at all, is negligible.

In principle, we could attempt to make use of the data to try to fix the actual value of the death rate. However, we are concerned that the paucity of data would not enable us to reliably infer the scale of a small death rate, even if present.

We have detailed this analysis in the supplementary materials which, following the comments of the reviewer, have been substantially revised. At the same time, we have incorporated the following narrative into the main text.
'In analyzing the data, we determined that the impact of crypt death is minimal (Sup. theory).'

The estimates of the crypt fission rate are significantly lower than previous direct estimates:
Li, et al. (1994). The crypt cycle in mouse small intestinal epithelium. Journal of cell science, 107 (Pt 12), 3271-3279.
Totafurno, J., Bjerknes, M., & Cheng, H. (1987). The crypt cycle. Crypt and villus production in the adult intestinal epithelium. Biophysical journal, 52(2), 279-294.
This should be discussed. The reviewer imagines that the fitting of a birth-death process could lead to larger estimates of the crypt fission rate.

We are grateful to the reviewer for bringing these papers to our attention. In both cases, these studies developed complementary biophysical modeling schemes to address the question of crypt growth and fission. Indeed, the fission rate estimated in this work is substantially lower than that reported in the early study, we find that our estimate is surprisingly consistent with the 1994 analysis which produced a fission rate for the WT crypt a factor of 3 larger than the one presented here. Given that this study was based on clonal marking using a mutagen, we believe that this level of coincidence is a 'triumph' rather than a discrepancy. We have now included a short discussion of this early work in the supplementary materials section.

4) The authors' study is predicated on the assumption that crypts follow a canonical crypt cycle that has a regular (average) duration. Do the authors consider that they have evidence for this hypothesis? Or is their data better represented by random induction of fission due to some local stimulus (eg no characteristic timescale for the life-cycle)?

To model the effect of crypt fission, we considered a Poisson random process in which the timing between consecutive fission events was chosen at random with an average set by 1/f and statistically uncorrelated (Markovian). In this sense, we did NOT assume that the crypts follow a canonical crypt fission cycle with a regular duration. Beyond the quantitative agreement of the predicted crypt clone size distribution with the data, we do not have direct evidence for the statistics of the crypt fission events. Indeed, it could be that crypt fission is triggered by a local stimulus, and that there is no characteristic timescale for the life-cycle beyond the average frequency of these stimuli. To acknowledge these points, we have now included the following narrative:

'Although we assumed that the timing between consecutive divisions is statistically uncorrelated, the exact mechanistic process that promotes crypt fission, such as an overload of stem cells and Paneth cells, is unknown [25]. In our hands, as well as previously documented, the Lgr5 stem cell pool, as well as average crypt size, remains roughly unaltered after mutating K-ras [31]. Alternatively, fission as a response to tissue injury might correlates with the non-uniform spreading of crypt fission. Analysis of human samples indeed suggests that fission is more common in crypts isolated from adenomas and hyperplastic polyps [32].'

Minor:

Abstract: The phrase "mutant tissue expands" is a bit funny. Just describe it as clonal expansion?

We have substantial rewritten the abstract and manuscript. Where appropriate, we have tried to refer to clonal expansion.
In the abstract: '…can initiate the expansion of such clones.' and '… mutant crypts clonally expand within the epithelium ….'

Concept of fitness in the introduction. You could be specific as to what phenotypes increase fitness. Eg., a proliferation or survival advantage, or something to do with spindle orientation.

We agree with the referee that there may be many mechanisms that contribute to the competitive advantage. Considering the known effect of oncogenic K-ras on the cell cycle, we have now performed experiments to address this point. In short, our experiments indicate that in K-ras mice, more CBC stem cells enter the S-phase during the EdU pulse. Considering no observed changes in the number of Paneth cells or the size of the Lgr5 CBC stem cell niche, this indicates that their cell cycle is faster. We therefore believe that the acceleration of cell cycle following oncogenic K-ras activation is the principle, if not the sole, reason for bias.

Results, section on Paneth cells in crypt fission. A p-value should be provided to justify the statement of equal numbers of Paneth cells in the two crypt branches. A binomial test is likely appropriate.

Our apologies. We did not wish to claim that the pre-existing Paneth cell niche was exactly divided in half. We have now changed the text that stated that indeed pre-existing Paneth cells are distributed to both new crypts.

'… we noticed distribution of pre-existing Paneth cells to both sides… .'

Referee #2:

Experimentally this is an elegant study that makes use of the possibility of labelling and tracing individual stem cells to study the effects of an oncogenic ras mutation in the stem cell compartment and at following stages. Results suggest that ras increases the rate of drift to clonality within the stem cell compartment and also increase the rate of crypt fission which would allow spreading of the oncogenic mutational in line with the concept of field cancerization. In fact this is what one would expect if ras endows cells with a growth advantage over wild type cells but it has not been studied before in a similar manner as here. Leaving aside the mathematics that needs to be reviewed by an expert, several points however are not clear to this reviewer because there are some gaps in the description of the study design and interpretation of results. Comments below should serve as suggestions to clarify certain points of this otherwise elegant study.

Unclear points

1 The scheme in Fig. 1 indicates that the loss of stem cells through differentiation and replacement by neighbouring stem cell leads to the domination of one stem cell over the others ultimately resulting in a clone of stem cells in a given crypt. If authors restrict their confocal investigations to the level of the stem cell compartment as suggested by the scheme why are Lgr5 high cells i.e. stem cells declining after tracing? Shouldn't all cells at this level be LGR5 positive? What is the nature of the other cells in the "clone"? Are they differentiated but still remain at this position of the crypt?

© European Molecular Biology Organization

We regret that our original narrative created some misunderstanding. First, in shortening the manuscript, we have transferred the scheme of the original figure 1 to the supplementary materials-section. As a result, we have been able to extend the figure and its legend in order to clarify the model.

In short: Cells that belong to a clone are now represented by the red Confetti color along the circumference. However, since these cells are still Lgr5 stem cells we maintained the 'body' of the cell in the color green to illustrate that the number of stem cells remains unaltered within the framework of the dynamics. We hope that these changes serve to clarify the nature of the model and thereby answer the questions that have been raised by the reviewer.

2 Related to point 1: The authors state that in the K-ras mice more Lgr5 positive cells "survive" than in the WT control. What is meant by surviving? Does it mean that ras prevents stem cells from developing progeny towards the transit amplifying compartment? Wouldn't that rather limit spreading of the mutation?

In normal homeostasis, Lgr5+ stem cells at the base of a wild-type crypt undergo neutral competition. In this process, cells that become displaced from the niche region loose stemness and progressively commit to a differentiation pathway. Following the marking of a single stem cell, its clonal progeny compete for niche access. As a result, the number of Lgr5+ cells within a single clone may increase or decrease over time until the entire stem cell compartment in the niche has become fully colonized or the clone has lost all Lgr5+ cells. In the course of this "neutral drift" dynamics, the displacement of cells from the niche constantly gives rise to cells in the transit-amplifying cell compartment. To discriminate clones that contain at least one Lgr5+ cell from those in which all cells have committed to a differentiation pathway, we designate the former as "surviving". Since, in the process of neutral competition, any Lgr5+ cells may eventually colonize the crypt, every surviving clone has a chance to fully colonize a crypt.

Following K-ras activation, we find that mutant cells experience a survival advantage over their wild-type neighbors, i.e. the division of a K-ras mutant Lgr5+ cell is more likely to displace a wild-type Lgr5+ neighboring cell from the niche, with the attendant loss of stemness, than the reverse. In this sense, the K-ras mutant Lgr5+ cells experience a "survival" advantage over their wild-type neighbors. However, the survival advantage translates only to a bias, i.e. despite this advantage, wild-type cells can still by chance outcompete a K-ras neighbor. Furthermore, the bias is experienced only at the boundary between wild-type and K-ras mutant Lgr5+ stem cells. Within and outside a K-ras mutant clone, the competition is neutral (i.e. unbiased). Therefore, in the course of biased drift, cells are constantly "fed" into the transit-amplifying cell compartment both from within and outside the clones.

Following the analysis of cell kinetics using the incorporation of a thymidine analogue, we believe that much of the survival advantage of K-ras mutant stem cells, if not all, derives from an increase in the cell cycle time of K-ras mutant cells over their wild-type neighbors.

We hope that this discussion serves to clarify the basis of the theoretical modeling scheme and address the questions raised in the report. Following the remarks of the reviewer, we have also implemented changes to the text which we hope clarifies the model more clearly.

3 It is suggested that this study reveals in vivo insights to the field cancerization. This appears to be quite evident by the finding that in K-ras mice there is an increased rate of crypt fission which will allow the spreading of the mutation. However, at the levels of stem cells this is less clear because in the experimental setting all stem cells should have the mutant K-ras expressed and no competition between normal and mutated cell can take place. Thus; what is observed is a faster "drift to clonality" i.e. faster loss and replacement among K-ras mutated cells. One cannot predict whether mutated stem cells would out-compete wild-type stem cells in a mosaic situation. If for instance K-ras acts in a non cell autonomous fashion, e.g. by secretion of growth factor it might also promote drift to clonality of wild type cells.

We agree with the referee that, in the event that all stem cells are mutant for K-ras, the intrinsic benefit towards neighbors, for instance a faster cell cycle, is "neutralized" since all stem cells obtain

© European Molecular Biology Organization

the same advantage. Such a situation is considered to be neutral again, because the stem cell pool remains homogenous and each stem cell has the same chance to colonize the crypt. Indeed, with an accelerated cell cycle time, the drift to monoclonality will be proportionately accelerated.

Importantly, in our experimental settings, we do analyze K-ras mutant stem cells that outcompete wild-type stem cells in a mosaic situation since we only activated mutant K-ras in a sporadic manner in single Lgr5 stem cells. These mutant stem cells are subsequently marked using the R26R-Confetti allele for clonal analysis, while the wild-type neighboring stem cells (also expressing Lgr5-EGFP) remain unmarked by R26R-Confetti.

Whether K-ras acts in a non-cell autonomous fashion, e.g. by secreting growth factors, can formally not be excluded but can, in our opinion, be neglected. In particular, secretion of growth factors would affect most stem cells in the same crypt in the same manner, thereby equaling out its effect over mutant vs wild-type. Since we score a clear competitive advantage for K-ras mutant stem cells over wild-type stem cell, we strongly believe these effects are driven by intracellular mechanisms.

4 In that context what is meant by the term "survival probability" of a K-ras mutant cell mentioned on p. 5 first line?

See the remarks for question 2. In a neutral competition situation, each stem cell in the crypt has an approximately equal chance to displace its neighbors and successfully colonize a crypt. Due to mutation a cell can obtain characteristics that confer an advantage during the competition (creating a bias). In our manuscript, we define the survival probability as the chance that a (mutant) stem cell ultimately displaces all of its neighbors and fully colonizes a crypt.

Referee #3:

The concept of field cancerization is well known in epithelial cancers particularly the skin. In this report Snippert and colleagues suggest KRAS mutations lead to field cancerization in the intestinal epithelium.
Overall the experiments performed are well, the data analysed correctly and it is clear KRAS mutations have an advantage over wild type stem cells and then Kras mutations are further expanded by crypt fission.
The questions though that remain however is
1) is this relevant for human CRC, most data have shown the p53 mutations spread in colitis(the elegant work of the Wright group) there is very little evidence of spreading of KRAS mutations.

Indeed, the reviewer makes a valid point. The concept of field cancerization is 'the clonal expansion of a genetically altered, but morphologically normal looking clone of cells, that predispose the tissue for cancer development.' With respect to the last part of the concept, we agree that we cannot formally address this aspect. While we observe expansion of mutant stem cells, it is not established here whether K-ras activation confers a predisposition for cancer development. Therefore, following the comments of this and another referee, we have revised the narrative to be more cautious on this issue.

Important though with respect to the first part of the field cancerization concept, we do provide insights into how cells that look morphologically normal can initiate clonal expansion within tissues via the mechanism of biased drift. Moreover, since more and more tissues reveal homeostatic mechanisms that rely on population asymmetry (thus neutral competition between stem cells), our results can be considered in a much wider perspective.

Second, in the revised manuscript, we have included further data that reveal that, in large part, the competitive survival advantage of K-ras mutant stem cells is obtained through a faster cell cycle time. From a broader perspective, this means that virtually all mutations that increase competitive advantage, for example via faster cell cycle, are biased towards clonal expansion through biased drift. We therefore believe that this study is important as it offers new insights into how neutral drift phenomena can be subverted by mutations to expand in fields of mutant cells while not creating phenotypic abnormalities.

2) There is no mechanism here of how KRAS mutation does this. At least some mechanistic work

could be done ie if Lgr5CREER KRASG12D mice are treated with MEK inhibitors does this suppress the stem cell expansion.
It will be important for the authors to attempt to address these questions as the paper currently in my opinion is a little too descriptive.

To begin to address this issue, we have now included a study of proliferation kinetics using the incorporation of a thymidine analogue that indicates that K-ras mutant stem cells have a cell cycle time that is increased substantially over their wild-type neighbors. This increased cell cycle rate explains, at least in large part, the underlying mechanism by which K-ras mutant stem cells acquire a competitive survival advantage over wild-type stem cells.

Other points
1) The mutation of human CRC show APC/b-catenin mutations at 80% and KRAS mutations at 40%. If KRAS mutations do spread first one would expect that sequencing of different places of the the same tumours would show concurrent APC and KRAS mutations. Does published work show this (eg Work of the Wright group)?

The referee correctly remarks that virtually all adenomas and cancers in the intestine carry activating Wnt pathway mutations, while about 40% have additional K-ras mutations. Importantly, we do not experimentally challenge the current model of ordered mutations (first activating Wnt, then K-ras). Yet, we think that there might be additional scenarios in which K-ras mutations might have occurred first, prior to Wnt pathway mutations. Especially in the light of the fact that K-ras mutations increase the target size for subsequent Wnt pathway mutations (Schwitalla et al., Cell 2013), we believe that this is a plausible scenario. Clinical data indeed suggest that in certain cases, K-ras mutations might have spread first (Aivado et al., Chirurg 2000; Zhu et al. Cancer res. 1997). Moreover, we believe that our manuscript can be viewed from a more general perspective. Any mutation that increases the cell cycle time will impose a competitive advantage on the mutant stem cell and drive clonal expansion, at least towards crypt fixation.

2) Given the authors have done Lgr5 APCfl/fl do they see the same increase in clone sizes and crypt fission (over the 14 day timepoint) prior to adenoma formation.

This is a very interesting point. We have performed similar experiments for APC. Deletion of APC actually leads to immediate transformation of the tissue and the formation of rapidly growing adenomas within a week (Barker et al., Nature 2009). It is thus qualitatively different from activation of K-ras.

3) Is the same KRASG12D effect seen in the colon? A much greater effect of this allele is seem when it is targeted to the colon (Haigis paper Nature Genetics)

Indeed, several reports indicate that KrasG12D confers a hyperproliferative effect in the colon, as in many other tissues. It is likely to assume that due to hyperproliferation of the K-ras mutant clone in the colon crypts, it will also outcompete its wild-type neighbors. However, we deliberately chose to study the small intestine (duodenum and jejunum) since it was documented that mutant K-ras had no morphological phenotype in this organ upon induction in adult mice (Sansom et al). With respect to field cancerization, we wanted to induce an oncogenic mutation in the stem cell compartment, but importantly, without affecting the functionality and morphology of the mutant clone. Our principle interest is and was the manipulation of neutral drift dynamics as an underlying mechanism for the first events in ‘field cancerization’.

4) Better citing of the working where KRASG12D mutation has been targeted to the mouse intestine is required. For example data has already been published suggesting KRAS does not alter the stem cell pool
Feng Y, Bommer GT, Zhao J, Green M, Sands E, Zhai Y, Brown K, Burberry A, Cho KR, Fearon ER. Mutant KRAS promotes hyperplasia and alters differentiation in the colon epithelium but does not expand the presumptive stem cell pool.
Gastroenterology. 2011 Sep;141(3):1003-1013

To our knowledge this paper was already cited, but we made the reference more explicit.
‘In our hands, as well as previously documented, the Lgr5 stem cell pool, as well as average crypt

size, remains roughly unaltered after mutating K-ras [28].'

Interesting this paper shows that differentiation is altered as did the Haigis paper. This should be discussed at the very least. It is interesting to note that this paper also shows that KRAS mutation causes a loss of paneth cells over the long term. This again should be discussed. Could KRASG12D stem cells be more likely to stay as stem cells and not produce a secretory/paneth cells offspring? Discussion of this data is also important given the discussion of the paneth cells in the paper.

The Haigis paper only analyses the colon of mice where they observe hyperproliferation. In this study, we deliberately choose to analyze the small intestine, because our primary interest lies at manipulations of the neutral drift dynamics with mutations that do not visibly alter the functionality or morphology of the mutant clone.

The paper of Feng et al., also analyses colon, as well as distal intestine. Their phenotype in the colon indeed resembles the observations made by Haigis and colleagues. With respect to the small intestine, their cre driver only worked in distal parts of the small intestine (ileum), while our studies are mainly performed in the duodenum and jejunum. Moreover, the expression of their cre-drivers starts during an early stage in development.

Sansom et al., Ray et al and Luo et al. activated oncogenic versions of K-ras in adult mice and observed no phenotypic alterations in the gut. Following the advice of the referee, we have now included a more extensive discussion in the supplementary information section on the different phenotypes that are observed while studying the effects of K-ras in the gut.

5) Quantification for figure 1 should be provided on the figure with statistics.

We agree with the referee and have restyled the figures. Quantification for figure 1 is now in the same figure as the raw data. Some other figure panels were transferred to the supplementary materials to meet requirements of the journal on length and where they could be discussed with more clarity.

2nd Editorial Decision 05 November 2013

Thank you for your patience while we have reviewed your revised manuscript. I have now had time to go through your study in detail. As I mentioned and you will see below, all referees now support the publication of your work in EMBO reports. Referee 2 suggests a couple of minor modifications, and from an editorial standpoint, some additional minor issues need to be addressed.

I am therefore writing with an 'accept in principle' decision, which means that I will be happy to accept your manuscript for publication once these minor issues/corrections have been taken care of:

- Please address the two issues raised by referee 2

- Please reformat the references in the supplemental theory according to EMBO reports style. Unpublished observations should not be included in the reference list, but be refereed to with the names of all authors as unpublished observations (or personal communication if authors are different from those of this study).

- I would suggest to move supplementary figure 3 back into figure 4, as the legend is not too long, and it is more informative here.

- The materials & methods section needs to be expanded to allow the readers of the main text to follow all the experiments described (although details needed to reproduce experiments can be supplementary). I appreciate this will mean lengthening the text, please keep this within reason.

© European Molecular Biology Organization

- As a standard procedure, we edit the title and abstract of manuscripts to make them more accessible to a general readership. I have edited the title to make it more general, as this will attract a more general readership and the oncogenic mutation used is clearly stated in the abstract. Please find the edited versions below carefully and ensure they maintain the meaning. Let me know if you do NOT agree with any of the changes.

"Biased competition between Lgr5 intestinal stem cells driven by oncogenic mutation induces clonal expansion

The concept of 'field cancerization' describes the clonal expansion of genetically altered, but morphologically normal cells that predisposes a tissue to cancer development. Here, we demonstrate that biased stem cell competition in the mouse small intestine can initiate the expansion of such clones. We quantitatively analyze how the activation of oncogenic K-ras in individual Lgr5+ stem cells accelerates their cell division rate and creates a biased drift towards crypt clonality. K-ras mutant crypts then clonally expand within the epithelium through enhanced crypt fission, which distributes the existing Paneth cell niche over the two new crypts. Thus, an unequal competition between wild type and mutant intestinal stem cells initiates a biased drift that leads to the clonal expansion of crypts carrying oncogenic mutations."

- Starting in 2014, every paper will include a 'Synopsis' to further enhance its discoverability. Synopses are displayed on the html version and they are freely accessible to all readers. The synopsis includes a short standfirst text -I have added my proposal for this text below- as well as 2-4 one sentence bullet points that summarise the paper. These should be complementary to the abstract -i.e. not repeat the same text. We encourage inclusion of key acronyms and quantitative information. In addition, synopses have a 'visual summary' of the paper. Could you supply a scheme/image that is 550 pixels wide by 150-400 pixels high, and the bullet points to accompany the standfirst? Please use the passive voice. Do let me know if you would like to modify the standfirst blurb:

"The fate of normal intestinal stem cells is determined through neutral competition. This study shows that when oncogenic K-Ras mutations arise, biased stem cell competition leads to a drift towards mutant crypt expansion that could be the underlying cause of field cancerization.

2-3 bullet points"

After all remaining issues have been attended to, you will receive an official decision letter from the journal accepting your manuscript for publication in the next available issue of EMBO reports (likely February). This letter will also include details of the further steps you need to take for the prompt inclusion of your manuscript in our next available issue.

Your study will be included in our February issue, but will appear online ahead of print as soon as possible. This would normally be toward the end of November. However, we are in the midst of changing publisher and the 2014 articles cannot be posted online until December 15th. I apologize for this 2 week delay; I will ask that your study be fast-tracked and among the first ones to be published online with the new publisher.

I look forward to receiving the final version of your study.

REFEREE REPORTS:

Referee #1 (Report):

This paper has been revised appropriately within the limits of reasonable experimental possibilities and I would recommend that it be published.

Referee #2 (Report):

The authors have clarified the questions raised by this reviewer.

Minor suggestions
Probably due to length constraints the initial papragraph on "lineage tracing strategy" has been deleted and moved to the supplementary information. Authors should refer to the supplement in the main text.

Although probably obvious to most experts in the field the fact that K-ras was activated in a sporadic manner thus creating a mosaic suituation within wt cells is essential to the experimental strategy and should be briefly mentioned.

Referee #3 (Report):

In this revised manuscript the authors have addressed the majority of my comments.
1) They have softened the interpretation of the field cancerization work and have altered the title.
2) They have provided a better discussion of previous work.
3) They included some data to suggested the increase in KRAS mutant stem cells is due to an increased proliferative advantage.

I feel this manuscript is now acceptable for publication

2nd Revision - authors' response — 06 November 2013

I have already resubmitted the final version of our manuscript (EMBOR-2013-37799V3).

In short,
-I have addressed the minor comments raised by referee 2.
-References in sup. theory are restyled.
-Sup fig 3 is moved back into figure 4.
-The edited title and abstract are perfect, thanks.
-Standfirst text is perfect, thanks.
-Bullet points are added as well as a visual summary of the manuscript.

One last remark. This time I opted for open access publication. However, I was unable to download the 'license to publish' file that is related to open access publishing. I was hoping that you could help me out.

3rd Editorial Decision — 06 November 2013

I am very pleased to accept your manuscript for publication in the next available issue of EMBO reports. Thank you for your contribution to our journal.

Regarding your enquiry about the Open Access license to publish and payment form, I will send the necessary forms to you as soon as our new publisher (Wiley) provides us with the new forms. Many thanks for your patience.

As part of the EMBO publication's Transparent Editorial Process, EMBO reports publishes online a Review Process File to accompany accepted manuscripts. As you are aware, this File will be published in conjunction with your paper and will include the referee reports, your point-by-point response and all pertinent correspondence relating to the manuscript.

If you do NOT want this File to be published, please inform the editorial office within 2 days, if you have not done so already, otherwise the File will be published by default [contact:

emboreports@embo.org]. If you do opt out, the Review Process File link will point to the following statement: "No Review Process File is available with this article, as the authors have chosen not to make the review process public in this case."

Thank you again for your contribution to EMBO reports and congratulations on a successful publication. Please consider us again in the future for your most exciting work.
